# Breviscapine regulates the proliferation, migration, invasion, and apoptosis of colorectal cancer cells via the PI3K/AKT pathway

**DOI:** 10.1038/s41598-023-33792-x

**Published:** 2023-06-14

**Authors:** Pengfei Niu, Feng Liu, Fuming Lei, Jisheng Peng, Yanzhao Wang, Jun Zhao, Zhaoya Gao, Qingkun Gao, Jin Gu

**Affiliations:** 1 Department of Gastrointestinal Surgery, Peking University Shougang Hospital, No. 9, Jinyuanzhuang Road, The Shijingshan District, Beijing, 100144 China; 2 Beijing Viewsolid Biotechnology Co., Ltd., Beijing, 100195 China; 3 Department of Traditional Chinese Medicine, Peking University Shougang Hospital, Beijing, 100144 China

**Keywords:** Cancer, Molecular biology

## Abstract

Colorectal cancer (CRC) is ranked as one of the most common malignancies with a high death rate. It has been discovered that breviscapine can alter the progression and development of various cancers. Nevertheless, the function and mechanisms of breviscapine in CRC progression have not yet been described. The cell proliferation capacity of HCT116 and SW480 cells was assessed using the CCK-8 and EdU assays. Cell apoptosis was tested through flow cytometry, and cell migration and invasion were examined using the transwell assay. Moreover, protein expression was examined through a western blot. Tumor weight and volume were assessed using the nude mice in vivo assay, and the Ki-67 protein expression was verified through the IHC assay. This study discovered that an increased dose of breviscapine (0, 12.5, 25, 50, 100, 200, and 400 μM) gradually reduced cell proliferation and increased apoptosis in CRC. Additionally, breviscapine restricted the migration and invasion CRC cells. Moreover, it was revealed that breviscapine inactivated the PI3K/AKT pathway and inhibited CRC progression. Finally, an in vivo assay demonstrated that breviscapine restrained tumor growth in vivo. It affected the CRC cells’ proliferation, migration, invasion, and apoptosis through the PI3K/AKT pathway. This discovery may offer new insights into CRC treatment.

## Introduction

Colorectal cancer (CRC) is considered one of the most common forms of cancer, with increasing incidence and high mortality rates^1,2^. After many years of research, a comprehensive cure based on surgical operation has become the primary form of treatment for CRC^3,4^. Among the major forms of treatment, drug therapy has expanded rapidly, significantly ameliorating the prognosis of late-period and metastatic CRC patients^5,6^. Thus, exploring novel drugs for improving the diagnosis and treatment of CRC is of great importance.

Evidence from previous research suggests that Chinese drug monomers and their analogs possess antitumor functions and are the most crucial sources of antineoplastic agents. For example, the Chinese drug eriodictyol, inhibits the MAPK/GSK-3β/ZEB1 pathway and reduces the migration and invasion of glioblastoma cells^7^, while baicalin regulates the CDK6/FOXM1 axis in prostate cancer and restricts tumor growth^8^. In addition, while luteolin suppresses stemness and heightens chemosensitivity in breast cancer^9^, jatrorrhizine affects the Wnt/β-catenin signaling pathway in CRC and represses tumor growth and metastasis^10^. Finally, xanthohumol reduces hexokinases II-mediated glycolysis and suppresses CRC progression^11^.

Breviscapine, a flavonoid glycoside, is the primary extract of *Erigeron breviscapus*, which has diverse pharmacological applications, such as anti-inflammatory, antioxidant, and vascular protection^12,13^. The main active components of breviscapine are baicalein, 4, 5, and 6-tetrahydroxy flavone-7-glucoside acid^14^. Several studies have demonstrated that breviscapine possesses antitumor activity against various cancers. For instance, breviscapine regulates the miR-129-5p/ZFP91 axis and inhibits prostate cancer progression^15^. Furthermore, it modifies the PAQR4-mediated PI3K/AKT pathway in prostate cancer and limits tumor growth and metastasis^16^. Breviscapine also represses inflammation and ROS (Reactive oxygen species) generation to relieve CCl4-mediated liver injury^17^. In addition, it up-regulates miR-7 to restrain the tumorigenesis of non-small cell lung cancer^18^, and it exhibits antitumor activity in hepatocellular carcinomas^19^. However, the effects of breviscapine on CRC progression have remained unclear.

In this study, the role of breviscapine in CRC progression was investigated in detail. The findings indicated that breviscapine reduces cell proliferation, migration, and invasion and influences apoptosis in CRC by modulating the PI3K/AKT signaling pathway. This discovery offers a promising therapeutic agent that could ameliorate the treatment of patients with CRC.

## Methods

### Cell lines and cell culture

The standard intestinal epithelial cell line NCM460 and human CRC cell lines (HCT116 and SW480) were acquired from the cell bank of the Chinese Academy of Sciences (Shanghai, China). The cells were maintained in an RPMI1640 medium (Gibco, New York, NY, USA) with 10% fetal bovine serum (FBS) in a humidified incubator with 5% CO_2_ at 37 °C. The cells were treated with breviscapine (0, 12.5, 25, 50, 100, 200 μM (Widely, Wuhan, China)) and 5-FU (10 μM (MillporeSigma, St. Louis, MO, USA)). In addition, the PI3K/AKT pathway activator (740Y-P (5 μL; Absin, Shanghai, China)) was also used in this study.

### CCK-8 assay

Cell proliferation was assessed using the cell counting kit-8 (CCK-8) assay (Beyotime, Shanghai, China). First, the NCM460, HCT116 and SW480 cells (5 × 10^3^ cells/well) were placed in a 96-well plate with breviscapine (0, 12.5, 25, 50, 100 and 200 μM) treatment, and cultivated for 48 h. Then, CCK-8 solution (10 μL) was mixed into each well and incubated for another 2 h at 37 °C. Finally, the absorbance (at 450 nm) was determined using a microplate reader (BioTek Instruments, Inc., Winooski, VT, USA).

### EdU assay

The HCT116 and SW480 cells were incubated in the 96-well plates with breviscapine (0, 25, 50 and 100 μM) treatment, and then mixed with 5-ethynyl-20-deoxyuridine (EdU) solution (RiboBio, Guangzhou, Guangdong, China) for 2 h. Next, the cells were immobilized using 4% formaldehyde and 0.5% Triton X-100 mixed with glycine. After rinsing with PBS (phosphate buffer saline), the cells were mixed with 100 μL of the Cell-Light™ Apollo 488 stain kit and stained with DAPI (4′,6-diamidino-2-phenylindole) (Beyotime, Nantong, China). After that, the EdU-positive cells were counted using fluorescence microscopy (Leica, Wetzlar, Germany).

### Flow cytometry

The Annexin V-FITC Apoptosis Detection Kit (Beyotime, Shanghai, China) was utilized in measuring apoptosis. First, the HCT116 and SW480 cells (1 × 10^3^ cells/well) were placed in 24-well plates and cultivated for 48 h. Then, the cells were harvested and resuspended in the binding buffer (100 µL), including Annexin V-FITC (5 µL) and PI (propidiumiodide) (5 µL), and incubated in the dark for 15 min. Finally, apoptosis was analyzed using flow cytometry (BD Biosciences, Franklin lakes, NJ, USA).

### Western blot

HCT116 and SW480 cells (1 × 10^3^ cells/well) were placed in 24-well plates and cultivated for 48 h, and then the cells were lysed with the RIPA lysis buffer. First, the isolated proteins were separated using SDS-PAGE and moved to the PVDF membranes. After sealing with non-fat milk, the membrane was co-incubated with primary antibodies, including proliferating cell nuclear antigen (PCNA) (ab92552, 1:1000, Abcam), cleaved-caspase three (ab2302, 1:1000, Abcam), p53 (ab28, 5 µg/mL, Abcam), N-cadherin (ab18203, 1:1000, Abcam), E-cadherin (ab40772, 1:1000, Abcam), p-PI3K (ab182651), 1:1000, Abcam), PI3K (ab191606, 1:1000, Abcam), p-AKT (ab8933, 1:500, Abcam), AKT (ab8805, 1:500, Abcam) and β-actin (ab8226, 1 µg/mL, Abcam) at 4 °C for 12 h. Next, the membranes were mixed with a secondary antibody (ab6721, 1: 2000, Abcam) for 1 h. Finally, the protein bands were analyzed using the ECL chemiluminescent detection system (Thermo Fisher Scientific, Rochester, NY, USA)^20,21^.

### Transwell assay

Some transwell chambers (8 µM pore size, Corning Inc) were coated with 50 µg Matrigel (BD, Biosciences), and others were without Matrigel. These chambers were utilized in the detection of cell migration and invasion. First, serum-free RPMI1640 (200 µL) cells (1 × 10^4^) were placed in the upper chamber. Then, the bottom chamber was filled with RPMI1640, which has 20% FBS. After 24 h, the transferred cells were fixed and stained using crystal violet. Lastly, the migrated or invaded cells were observed using a microscope; the migrated and invaded cells on the membrane’s lower surface were counted using three random fields.

### Wound healing assay

HCT116 and SW480 cells were grown to 80–90% confluence in six-well plates. Then, cell scratching was performed using a sterile pipette tip (10 µL). The scratch distance was observed under a microscope at 0 h and after 24 h.

### Nude mice in vivo assay

The male BALB/c nude mice (4–6 weeks old, n = 12) were procured from the Vital River Laboratory Animal Technology Co., Ltd. (Beijing, China). HCT116 cells (5 × 10^6^) were injected subcutaneously into the flank region of the nude mice. When the tumors reached approximately 5 mm in diameter, the intraperitoneal administration of breviscapine (40 mg/kg) or 5-FU (25 mg/kg) was performed^22^. The control group was treated with an equal dose of DMSO. After 2 weeks, the mice were killed using an intraperitoneal injection with an overdose of pentobarbital. Importantly, all the animal experiments were approved by the Ethics Committee of Beijing Viewsolid Biotechnology Co. LTD (VS212601467) and were performed per the relevant guidelines and regulations. Furthermore, this study is reported in accordance with the ARRIVE guidelines (https://arriveguidelines.org).

### IHC assay

After dewaxing with ethanol, the mice tumors’ tissue sections (4 μM) were mixed with H_2_O_2_ in methanol. Then, the sections were mixed with the primary antibody Ki-67 (ab15580, 1 µg/mL, Abcam) overnight at 4℃. After washing, secondary antibodies (ab6721, 1:1000, Abcam) were added and further incubated. Next, the sections were dyed with diaminobenzidine (DAB) and redyed with hematoxylin. Lastly, the images were observed using fluorescence microscopy (IX-51, Olympus).

### Statistical analysis

The data obtained from this study were presented as the mean ± standard deviation (SD). SPSS 20.0 software (SPSS Inc., Chicago, IL, USA) was used for statistical analysis and presented using GraphPad Prism Software 8.0. In addition, the experimental procedures were repeated at least three times. Finally, any observed group variation was presented using the Student’s t-test or a one-way analysis of variance (ANOVA) with the Tukey–Kramer multiple comparisons test. The results were considered statistically significant when *p* < 0.05.

### Ethical approval

All animal experiments were approved by the Ethics Committee of Beijing Viewsolid Biotechnology Co. LTD (VS212601467) and all experiments were performed per relevant guidelines and regulations. Furthermore, the study is reported per the ARRIVE guidelines (https://arriveguidelines.org).

## Results

### Breviscapine regulated the proliferation and apoptosis of CRC cells

**Figure 1:**
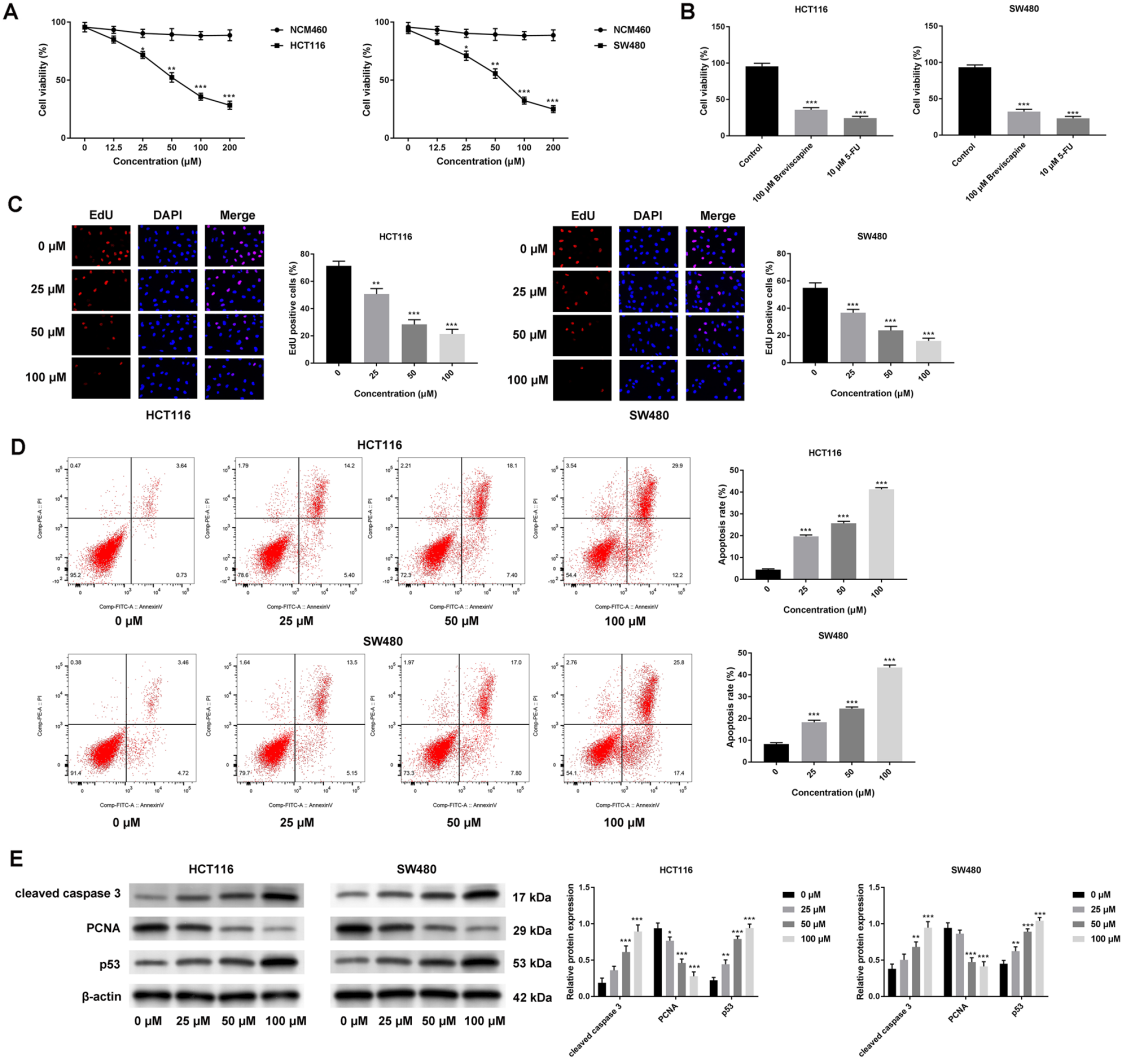
Breviscapine regulated the proliferation and apoptosis of CRC cells. (**A**) The cell viability of NCM460, HCT116 and SW480 cells was observed using the CCK-8 assay with different breviscapine concentrations (0, 12.5, 25, 50, 100 and 200 μM). (**B**) The cell viability of HCT116 and SW480 cells was examined through CCK-8 assay with breviscapine (100 μM) and 5-FU (10 μM) treatments. (**C**) The EdU-positive cells were assessed using the EdU assay in HCT116 and SW480 cells treated with different breviscapine concentrations (0, 25, 50, and 100 μM). (**D**) Apoptosis was verified through flow cytometry with different breviscapine concentrations (0, 25, 50, and 100 μM). (**E**) The protein expression of PCNA, cleaved-caspase three, and p53 was examined through a western blot with different breviscapine concentrations (0, 25, 50, and 100 μM). *p < 0.05, **p < 0.01, ***p < 0.001 vs 0 μM.

Breviscapine has been investigated regarding the development of many cancers, but its function in CRC is unclear. As displayed in Fig. 1A, the cell proliferation of HCT116 and SW480 was inhibited through breviscapine treatment using a dose-increasing technique, and breviscapine and 5-FU treatments both impaired cell viability (Fig. 1B). Further experiments using breviscapine (0, 25, 50, and 100 μM) were conducted in this study. Additionally, an EdU assay was performed to assess cell proliferation, and it was discovered that the EdU-positive cells (indicating cell proliferation ability) decreased with an increase in breviscapine concentration (0, 25, 50, and 100 μM) (Fig. 1C). During flow cytometry, the lower left quadrant contained living cells, the upper left quadrant had dead cells, the upper right quadrant had late apoptotic cells, and the lower right quadrant contained early apoptotic cells. The rate of apoptosis (the upper right quadrant + the lower right quadrant) increased with an increase in the breviscapine concentration (Fig. 1D). In addition, the expression of the PCNA protein decreased, while cleaved-caspase three and p53 expressions increased with an increase in breviscapine concentration (Fig. 1E). Altogether, breviscapine restrained cell proliferation and accelerated apoptosis in CRC.

### Breviscapine attenuated the migration and invasion abilities of CRC cells in Transwell assays

**Figure 2:**
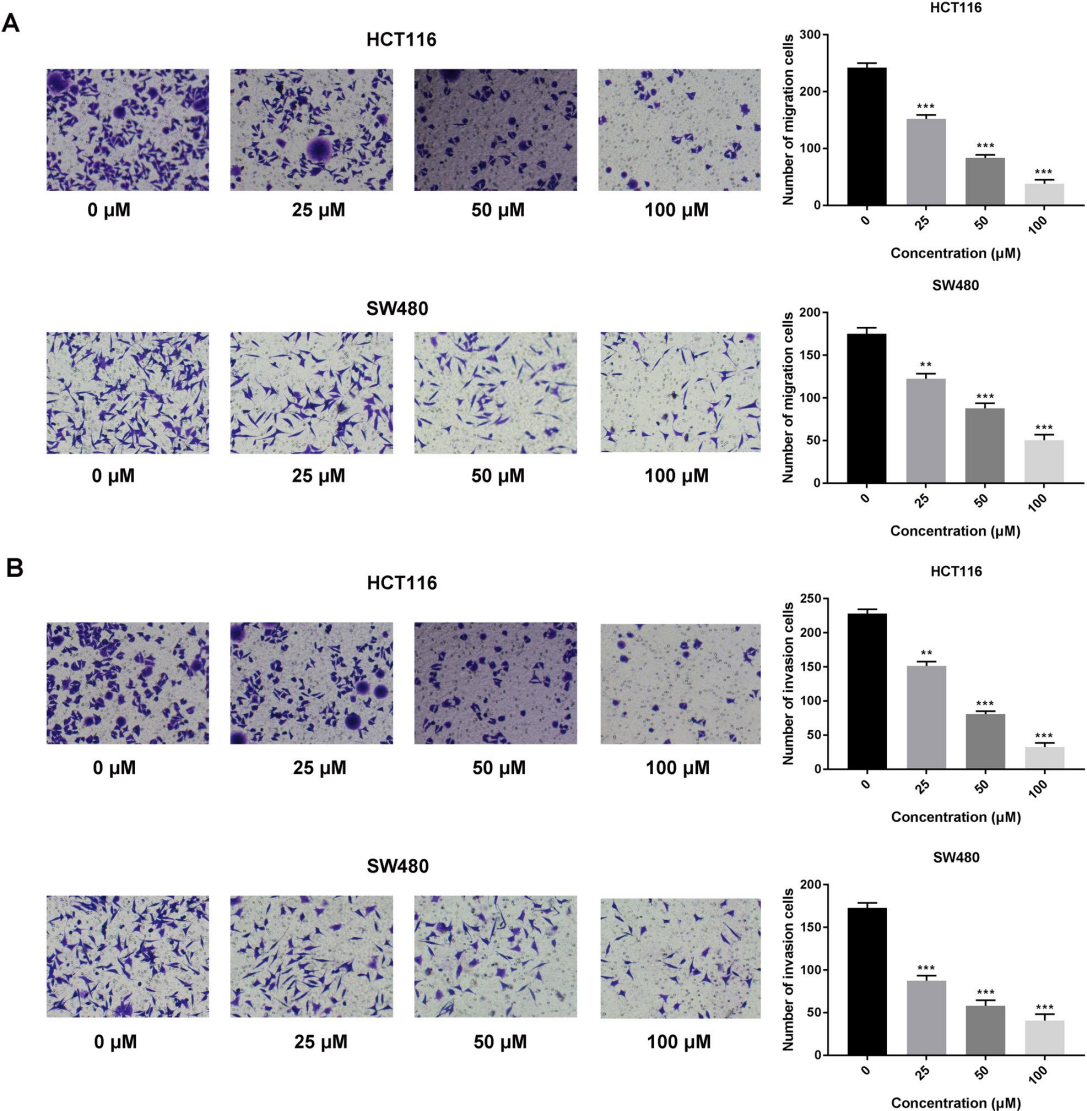
Breviscapine attenuated the migration and invasion abilities of CRC cells in Transwell assays. (**A**, **B**) The cell migration and invasion were evaluated through a transwell assay with different breviscapine concentrations (0, 25, 50, 100 μM). **p < 0.01, ***p < 0.001 versus 0 μM.

Next, through Transwell assays, it was demonstrated that cell migration and invasion were restricted with an increase in breviscapine concentration (Fig. 2A,B).

### Breviscapine inhibited cell migration and EMT process in CRC cells

**Figure 3:**
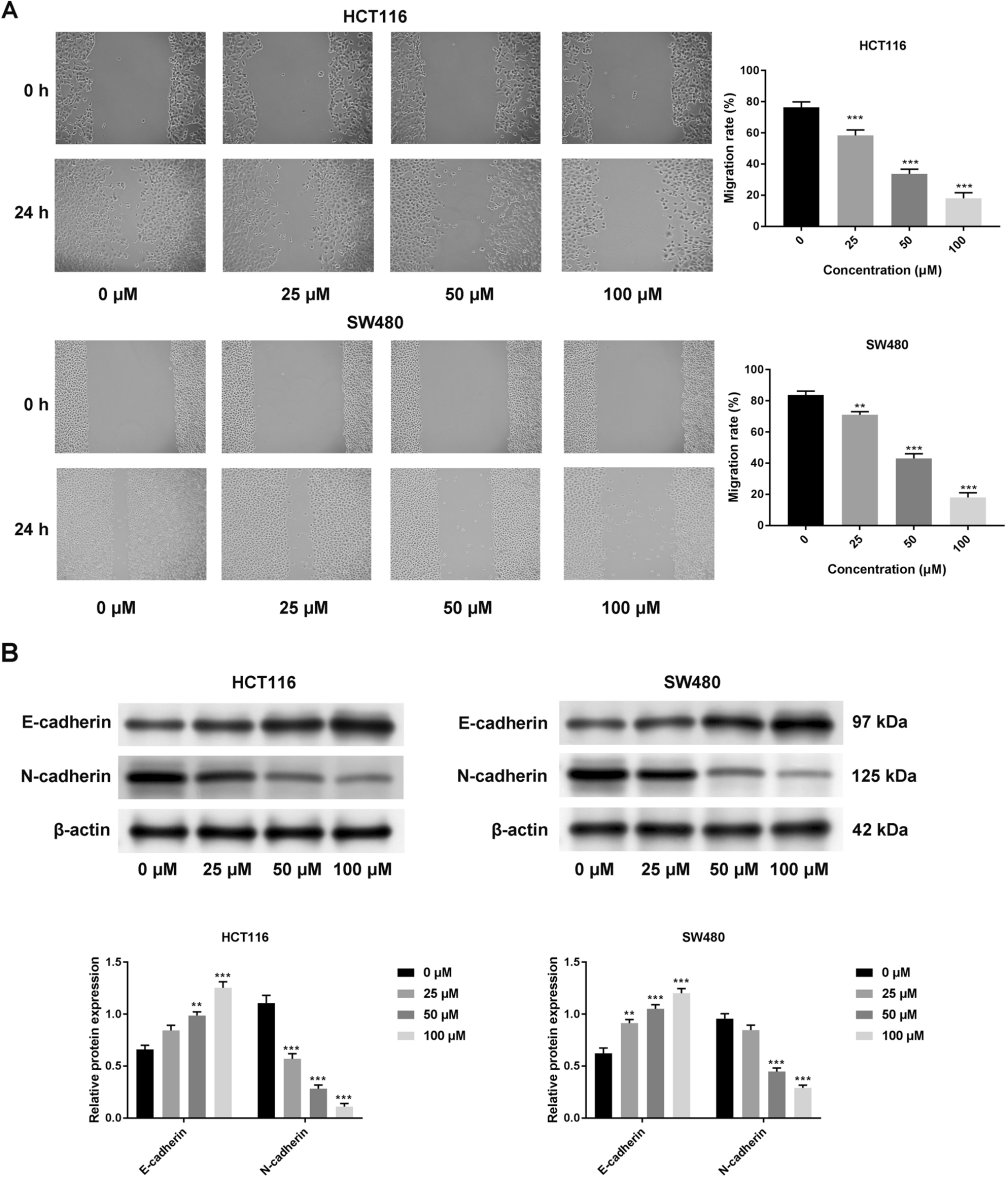
Breviscapine inhibited cell migration and EMT process in CRC cells. (**A**) The cell migration was measured with a wound-healing assay. (**B**) The protein expression of E-cadherin and N-cadherin was tested through a western blot with different breviscapine concentrations (0, 25, 50, and 100 μM). **p < 0.01, ***p < 0.001 versus 0 μM.

During the wound-healing assay, cell migration decreased after breviscapine treatment (Fig. 3A). At the same time, the EMT progress markers (the epithelial marker-E-cadherin and the mesenchymal marker-N-cadherin) were examined. The expression of N-cadherin was down-regulated, and E-cadherin expression was up-regulated with the increase in breviscapine concentration, indicating that the EMT process was restricted after breviscapine treatment (Fig. 3B). These results revealed that breviscapine limited the migration and invasion of CRC cells.

### Breviscapine regulated cell proliferation and apoptosis through affecting the PI3K/AKT pathway

**Figure 4:**
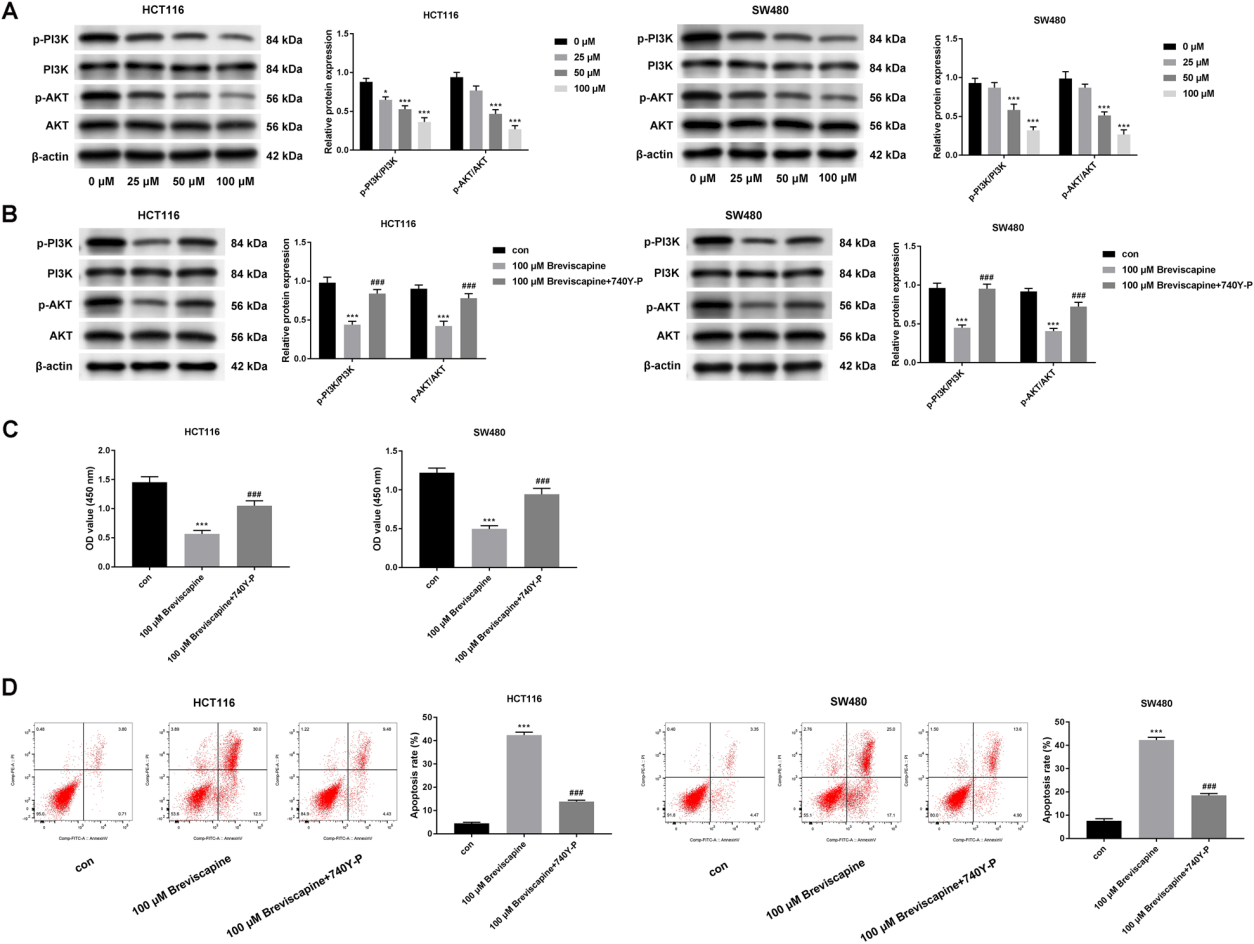
Breviscapine regulated cell proliferation and apoptosis through affecting the PI3K/AKT pathway. (**A**) The protein expression of p-PI3K, PI3K, p-AKT, and AKT were examined through a western blot with different breviscapine concentrations (0, 25, 50, and 100 μM). (**B**) The protein expression of p-PI3K, PI3K, p-AKT, and AKT was assessed through a western blot at 100 μM breviscapine, and 100 μM breviscapine + 740Y-P groups. *p < 0.05, **p < 0.01, ***p < 0.001 versus control; ^##^*p* < 0.01, ^###^*p* < 0.001 versus 100 μM breviscapine. (**C**) The cell viability was measured through CCK-8 assay at concentrations of 100 μM breviscapine and 100 μM breviscapine + 740Y-P groups. (**D**) Apoptosis was examined through flow cytometry at 100 μM breviscapine and 100 μM breviscapine + 740Y-P groups.

The PI3K/AKT pathway has been revealed to be a key regulator in CRC progression. Thus, breviscapine regulation of the PI3K/AKT pathway in CRC progression was investigated. The levels of p-PI3K and p-AKT decreased with the increase in breviscapine concentrations (Fig. 4A), while PI3K and AKT levels did not change. Moreover, it was discovered that the decreased p-PI3K and p-AKT levels facilitated by breviscapine (100 μM) were recovered after treatment with the PI3K/AKT activator (740Y-P) (Fig. 4B), and the reduced cell proliferation triggered by breviscapine was offset after 740Y-P treatment (Fig. 4C). In addition, apoptosis increased with breviscapine treatment; however, this change can be reversed after adding 740Y-P (Fig. 4D).

### Breviscapine suppressed cell migration, invasion and EMT progress through affecting the PI3K/AKT pathway

**Figure 5:**
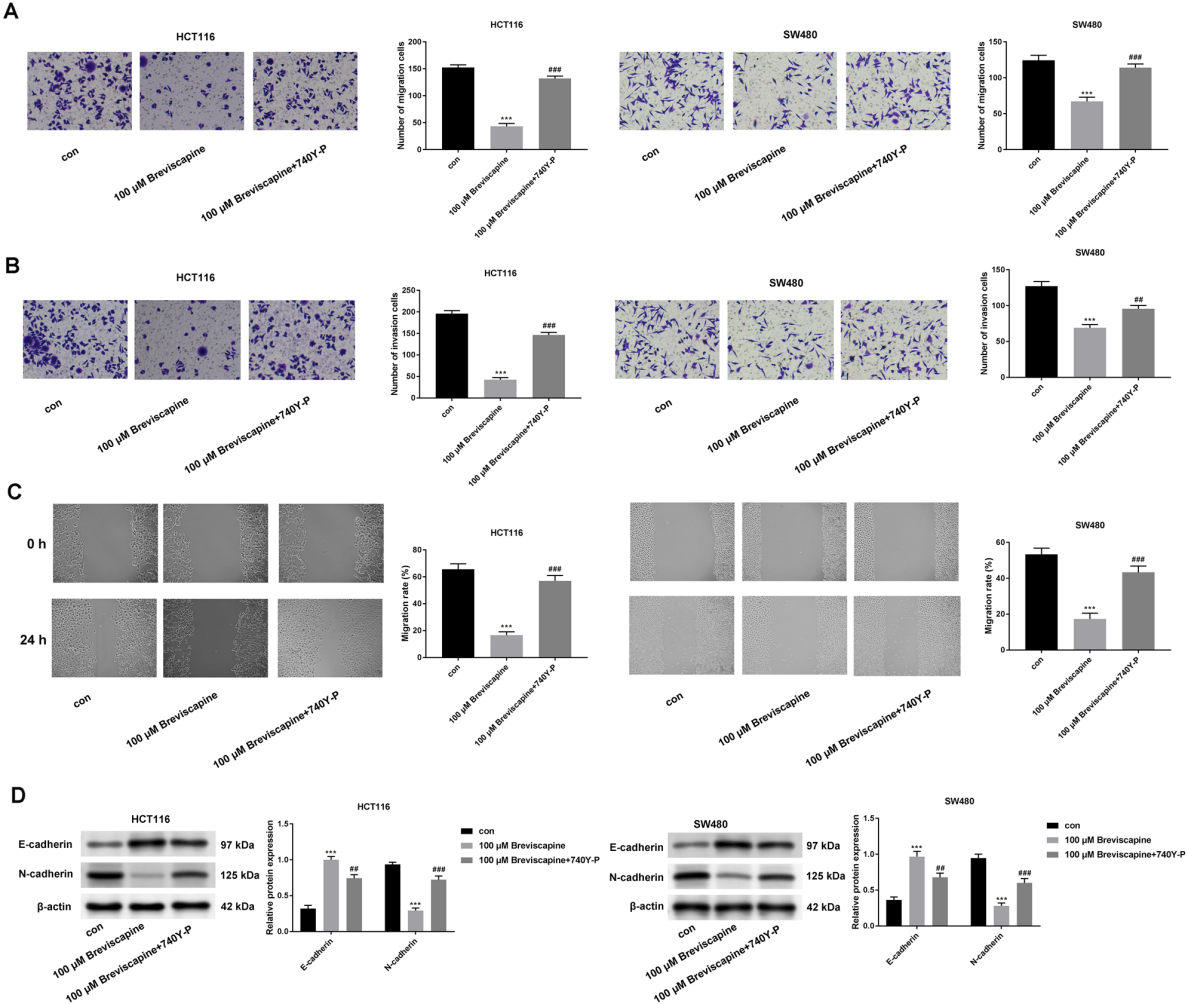
Breviscapine suppressed cell migration, invasion and EMT progress through affecting the PI3K/AKT pathway. (**A**–**C**) The cell migration and invasion were assessed through transwell and wound-healing assays at 100 μM breviscapine and 100 μM breviscapine + 740Y-P groups. (**D**) The protein expression of E-cadherin and N-cadherin was evaluated through western blot at 100 μM breviscapine and 100 μM breviscapine + 740Y-P groups. ***p < 0.001 versus control; ^##^*p* < 0.01, ^###^*p* < 0.001 versus 100 μM breviscapine.

Furthermore, the decreased cell migration and invasion induced by the breviscapine treatment can be countered by adding 740Y-P (Fig. 5A–C). Similarly, the down-regulated N-cadherin expression and the up-regulated E-cadherin expression induced by the breviscapine treatment can be neutralized after treatment with 740Y-P (Fig. 5D). In conclusion, breviscapine inactivated the PI3K/AKT pathway and inhibited CRC progression.

### Breviscapine inhibited tumor growth in vivo

**Figure 6:**
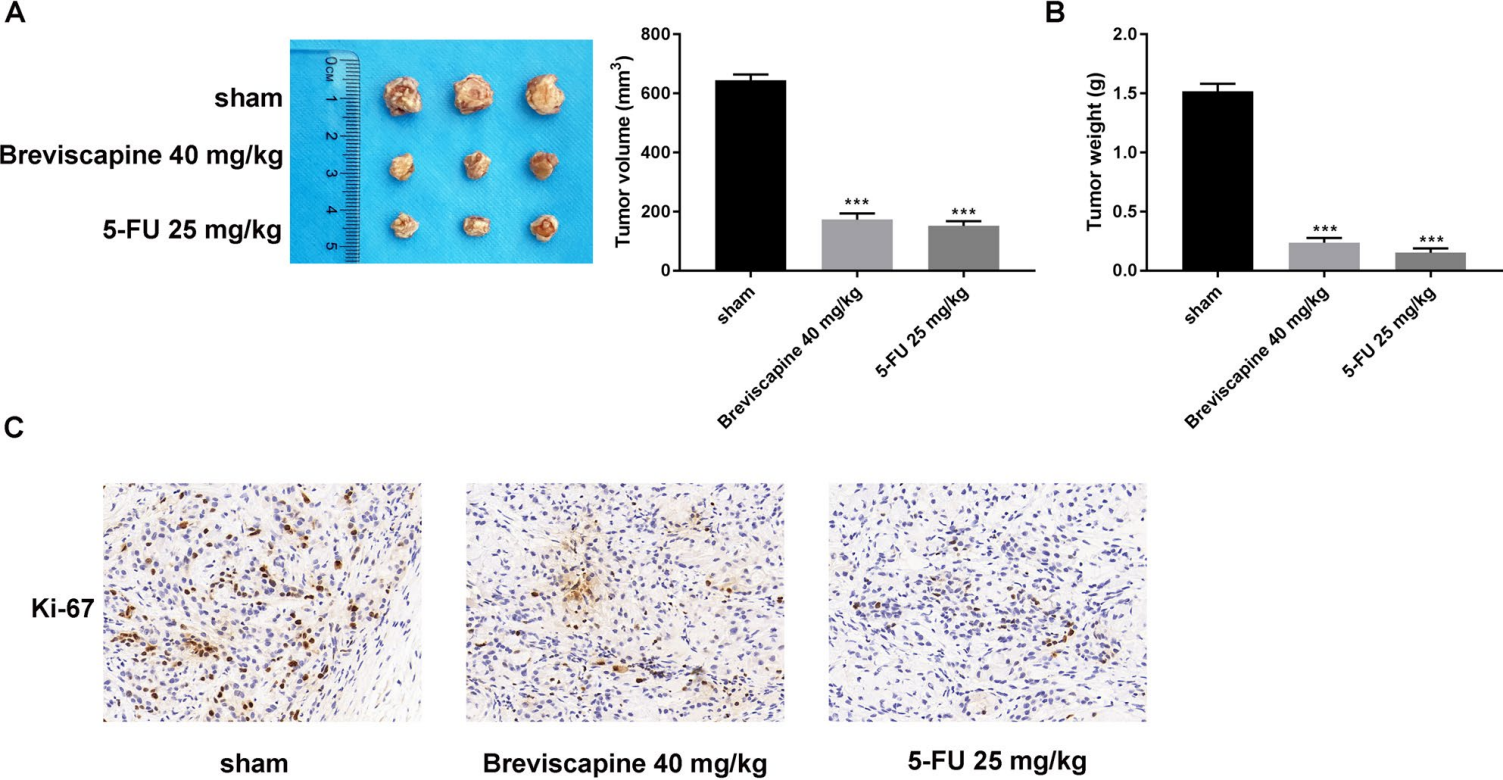
Breviscapine retarded tumor growth in vivo. (**A**, **B**) Tumor weight and volume were assessed in the control with the breviscapine 40 mg/kg and 5-FU 25 mg/kg groups. (**C**) The Ki-67 protein expression was validated through IHC assay in the control, breviscapine 40 mg/kg, and 5-FU 25 mg/kg groups. ***p < 0.001 versus control.

Further investigation in vivo indicated that the tumor weight and volume decreased after breviscapine (40 mg/kg) treatment (Fig. 6A,B). In addition, the IHC assay revealed that protein expression of the cell proliferation marker, Ki-67, was lower in the breviscapine 40 mg/kg group (Fig. 6C). These findings indicated that breviscapine inhibits tumor growth in vivo.

## Discussion

Due to the advancements in phytochemistry, multiple nontoxic and effective components have been isolated from plants, which offer a broad alternative resource base for developing novel antitumor drugs^23,24^. For example, breviscapine derived from *E. breviscapus* is one such natural flavonoid. Several studies have demonstrated that breviscapine has various pharmacological applications. For instance, it offers anti-inflammatory, renal, cardiovascular, and neural protection. Moreover, breviscapine regulates the PI3K/Akt/GSK-3β pathway and suppresses inflammation and myocardial apoptosis after coronary microembolization^25^. In addition, it exhibits antioxidant and anti-inflammatory properties that improve cognitive impairments triggered by transient cerebral ischemia and reperfusion^26^. Furthermore, breviscapine affects the Nrf2 signaling pathway and modulates IL-6 expression, thereby displaying neuroprotective effects and relieving traumatic brain injury^27,28^.

It has generally been recognized that breviscapine has antitumor properties^15–19^; however, its function in CRC remains unclear. Compared to previous research, it was discovered in this study that breviscapine regulates the proliferation and apoptosis of CRC cells and restricts tumor growth in vivo.

Cancer metastasis is a complex process in which tumor cells break away from the primary tumor, settle, and growth in other locations in the body^29,30^. Whether early or late, many cancer patients may eventually become metastatic^31^. Unfortunately, the current treatment methods have difficulty curing the metastatic lesions of distant organs, which results in the death of about 90% of cancer patients^32,33^. Thus, tumor metastasis is the principal challenge regarding cancer-related death^33^. Therefore, exploring the mechanisms of metastasis and seeking effective bio-targets for tumor metastasis to develop efficient treatment methods is inevitable.

It has been discovered that the PI3K/AKT pathway is stimulated in various cancers, strengthening tumor growth and metastasis. For example, SLC1A3 modulates the PI3K/AKT pathway, which facilitates gastric cancer progression^34^, while LncRNA HAND2-AS1 suppresses the PI3K/Akt pathway in non-small-cell lung cancer. In addition, it reduces cell proliferation and increases apoptosis^35^. While rotenone regulates the PI3K/AKT pathway to restrict colon cancer cell proliferation, motility, and EMT progress^36^, palmitic acid inactivates the pathway in prostate cancer to repress cell proliferation and metastasis^37^. Notably, breviscapine has been discovered to modulate the PAQR4-mediated PI3K/AKT pathway while inhibiting prostate cancer cell growth and metastasis^16^. However, the relationship between breviscapine and the PI3K/AKT pathway has not yet been investigated for CRC. This study revealed that breviscapine inactivates the PI3K/AKT pathway to inhibit CRC progression.

In conclusion, this study demonstrated that breviscapine weakened cell proliferation, migration, invasion, and apoptosis in CRC via the PI3K/AKT pathway. This may shed light on the role of breviscapine in improving CRC treatment. Nevertheless, this study had limitations, including the inability to explore angiogenesis, immune responses, exosome, glycolysis, and oxidative stress. Therefore, more experiments about the effect of breviscapine on other aspects of CRC progression will be conducted in the future (Supplementary information showed the raw data).

## Supplementary Information

Supplementary Information.

## Data Availability

All the data used to support the findings of this study are included in the article.
