# Supplementary figures and images for: Breviscapine regulates the proliferation, migration, invasion, and apoptosis of colorectal cancer cells via the PI3K/AKT pathway
Source: Sci Rep. 2023 Jun 14;13:9674. doi: 10.1038/s41598-023-33792-x (PMC10267214; doi:10.1038/s41598-023-33792-x)

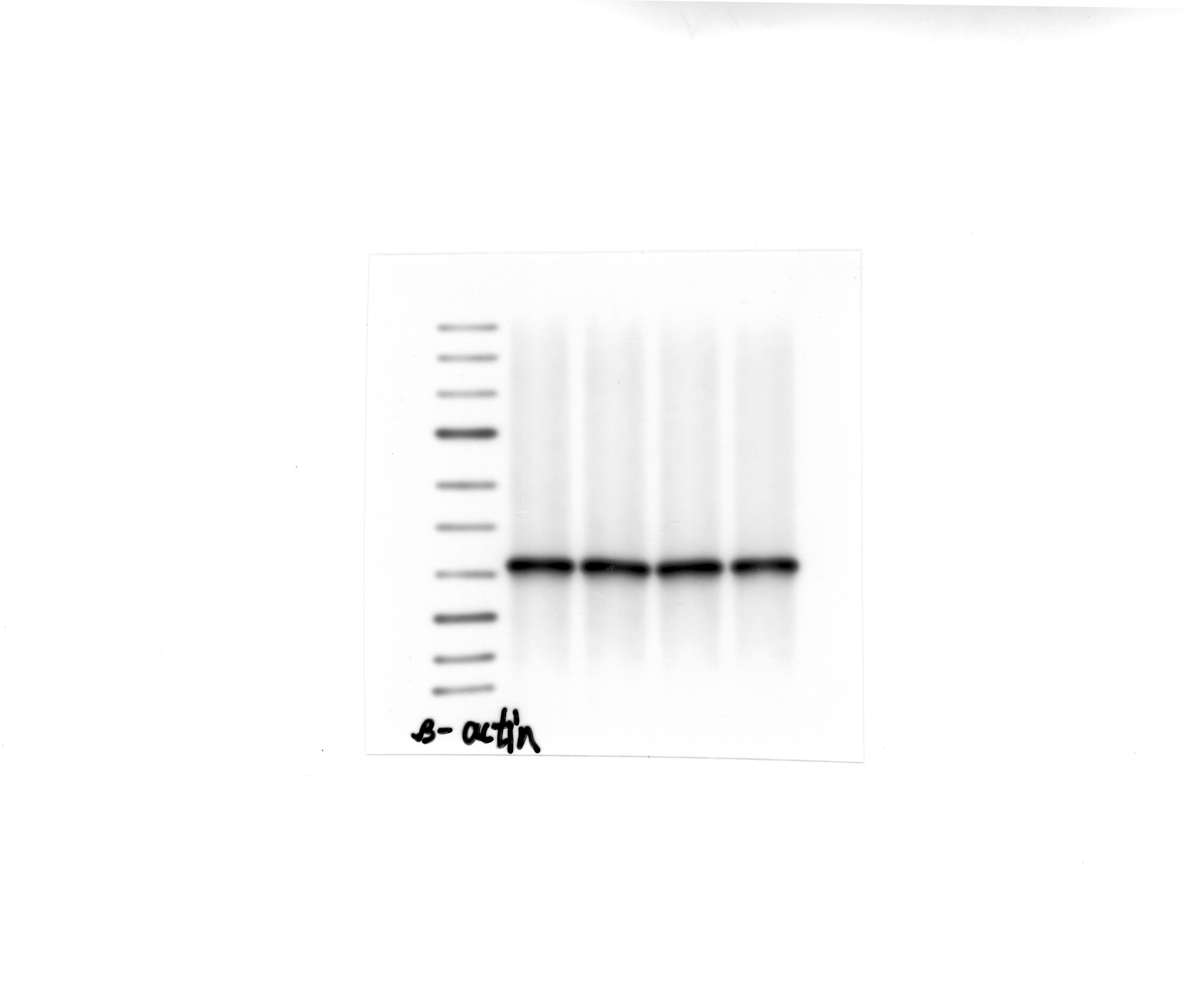

Supplement: Supplementary file 1 — Supplementary Information. [file 41598_2023_33792_MOESM1_ESM.zip › WB/fig 1E-HCT116/Actin.tif]

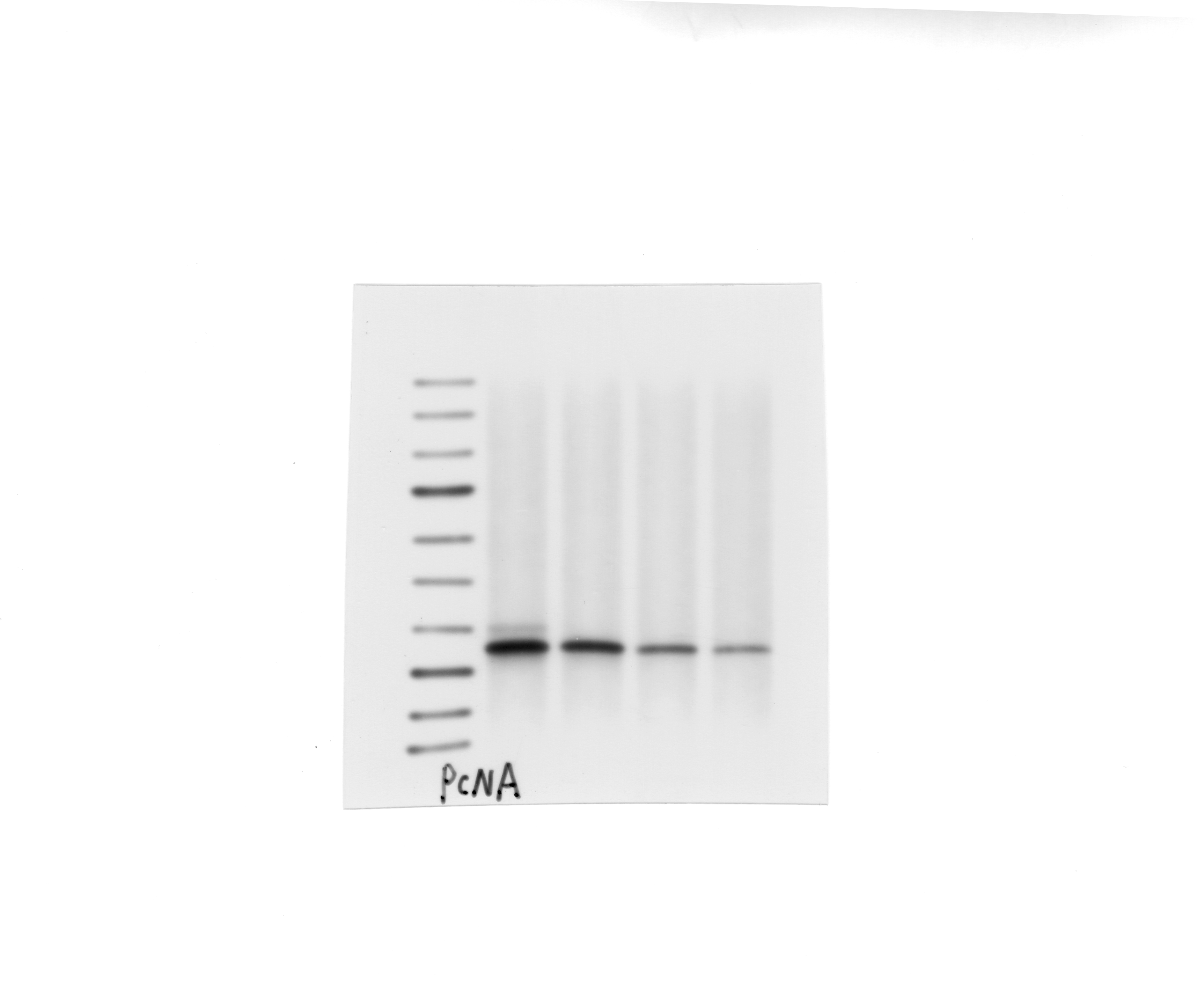

Supplement: Supplementary file 1 — Supplementary Information. [file 41598_2023_33792_MOESM1_ESM.zip › WB/fig 1E-HCT116/PCNA.tif]

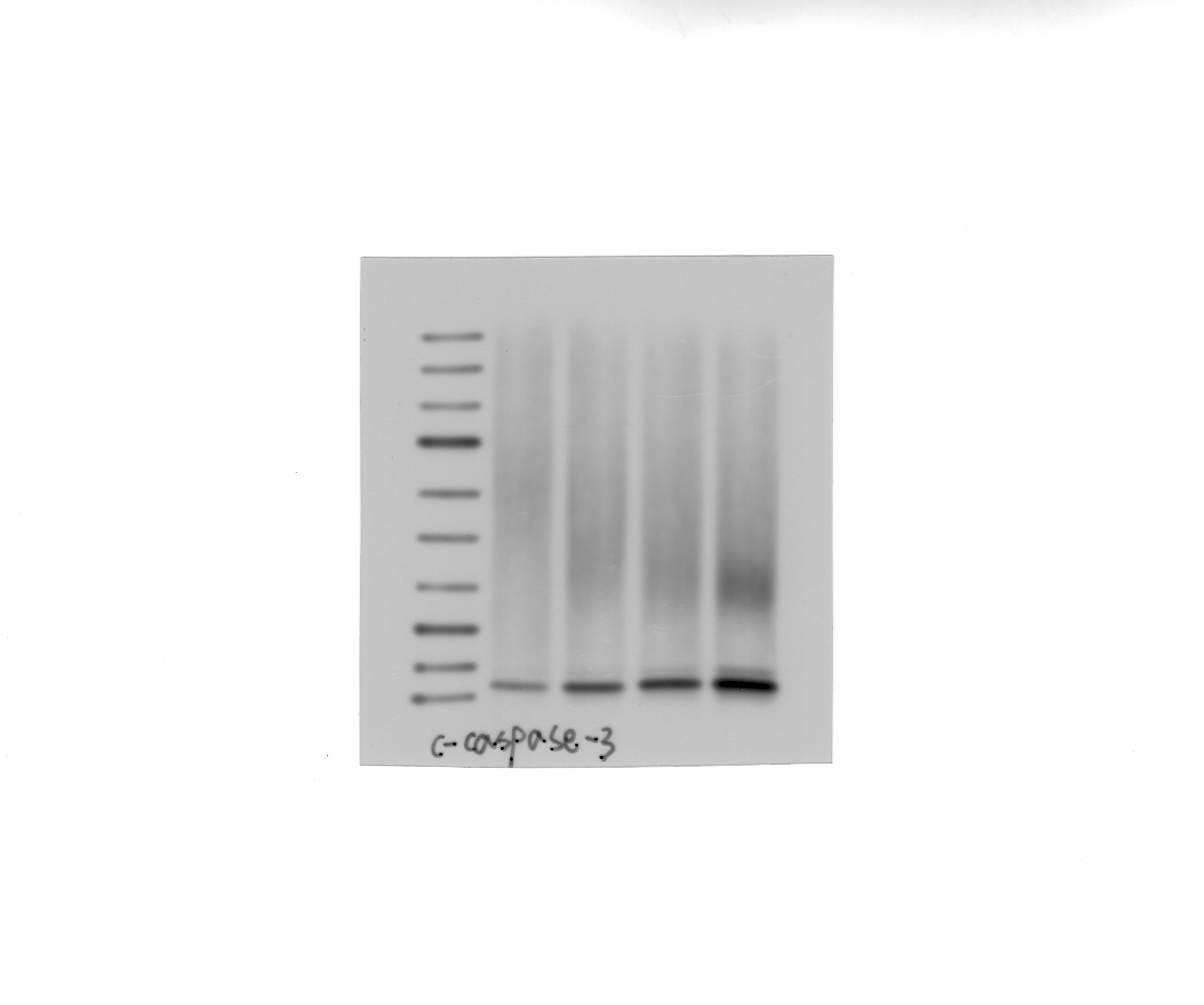

Supplement: Supplementary file 1 — Supplementary Information. [file 41598_2023_33792_MOESM1_ESM.zip › WB/fig 1E-HCT116/cleaved caspase 3.tif]

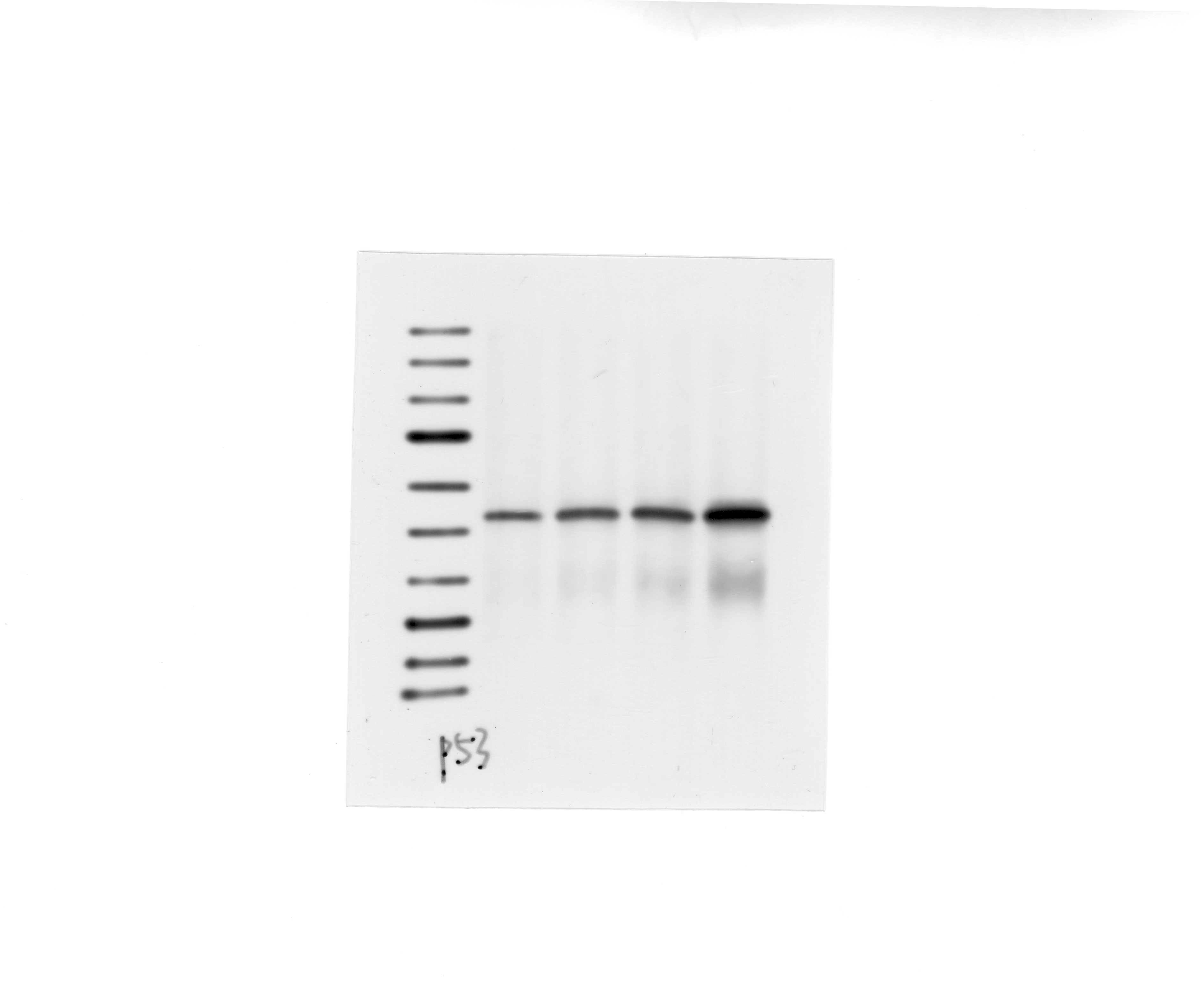

Supplement: Supplementary file 1 — Supplementary Information. [file 41598_2023_33792_MOESM1_ESM.zip › WB/fig 1E-HCT116/p53.tif]

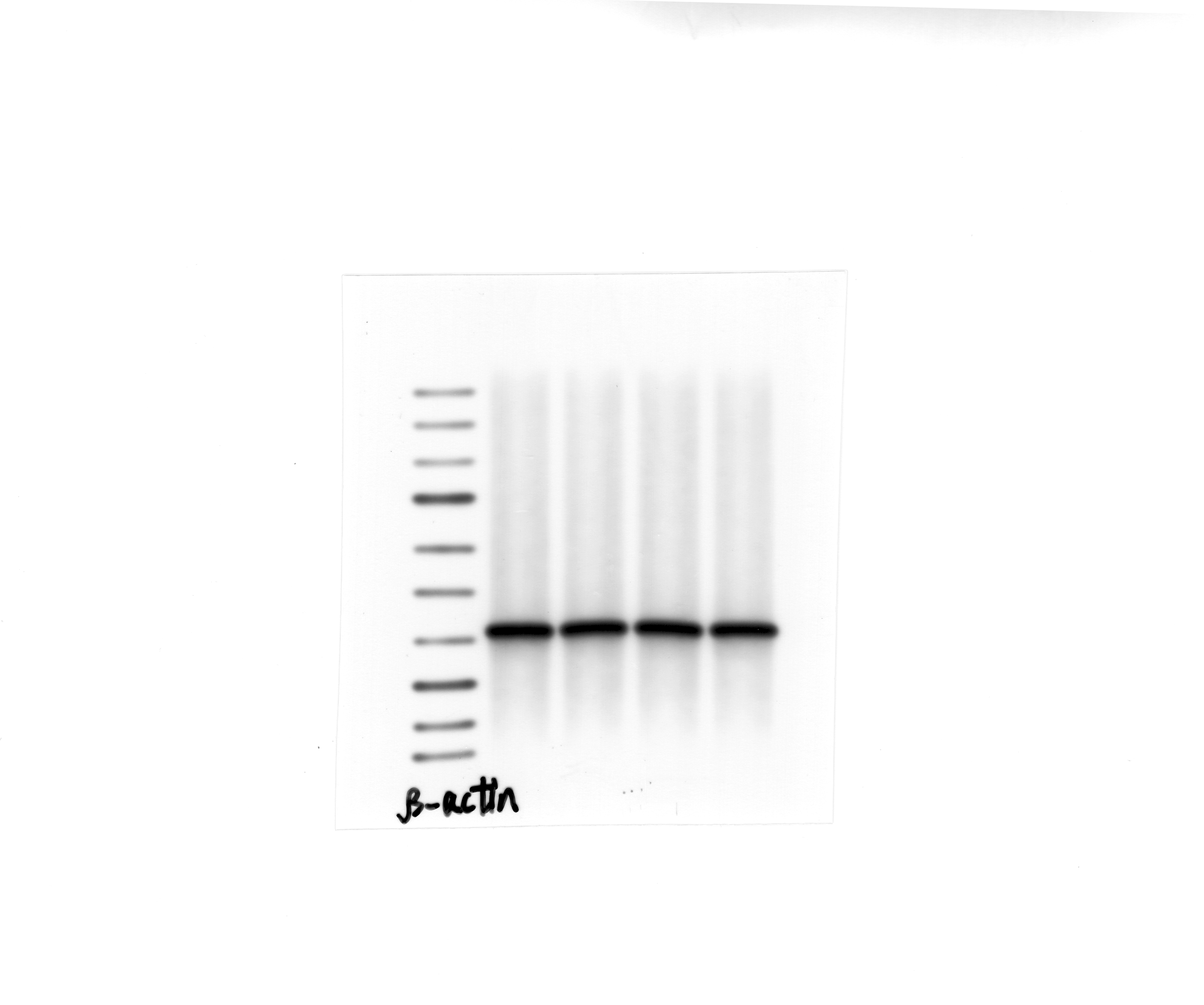

Supplement: Supplementary file 1 — Supplementary Information. [file 41598_2023_33792_MOESM1_ESM.zip › WB/fig 1E-SW480/Actin.tif]

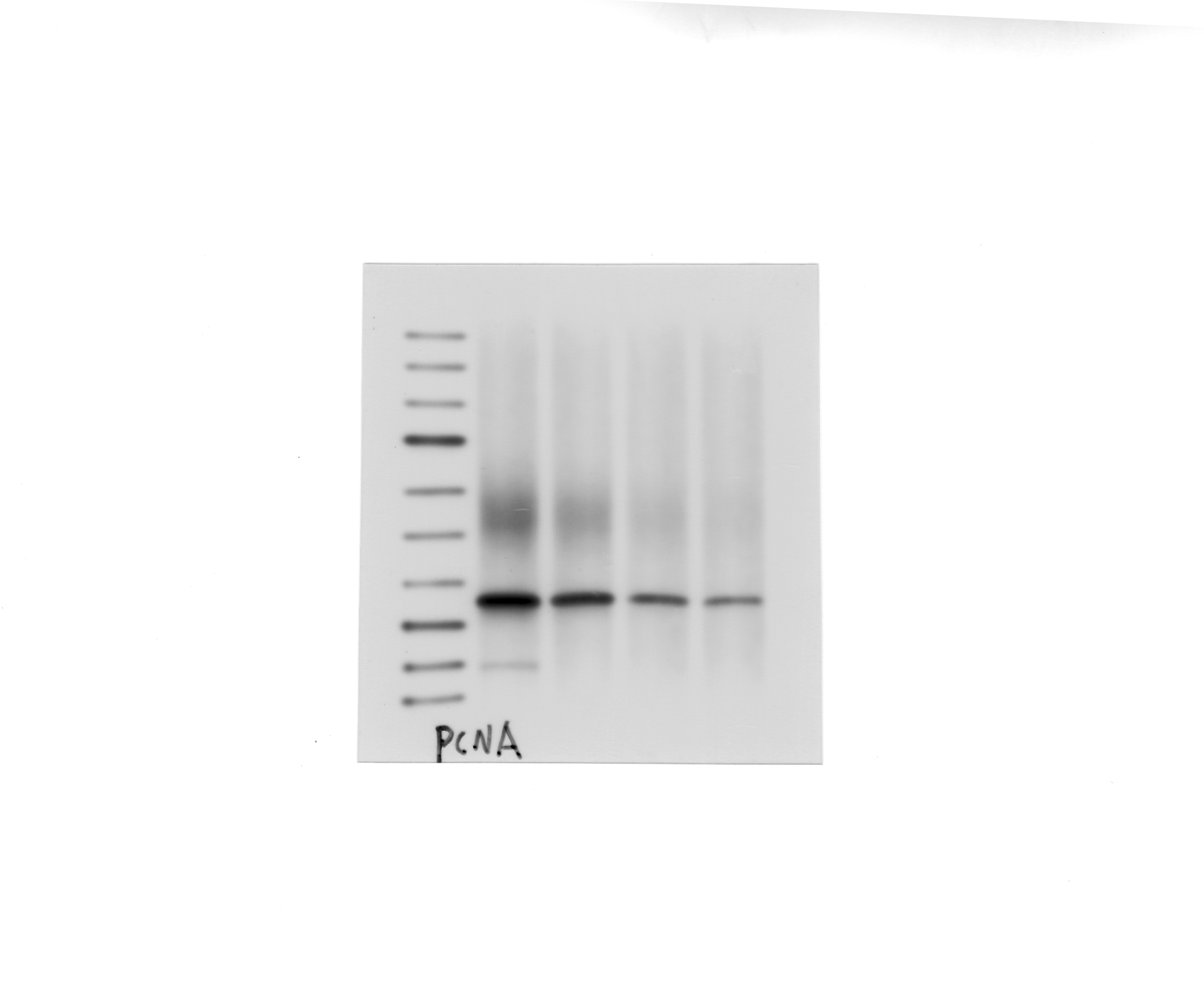

Supplement: Supplementary file 1 — Supplementary Information. [file 41598_2023_33792_MOESM1_ESM.zip › WB/fig 1E-SW480/PCNA.tif]

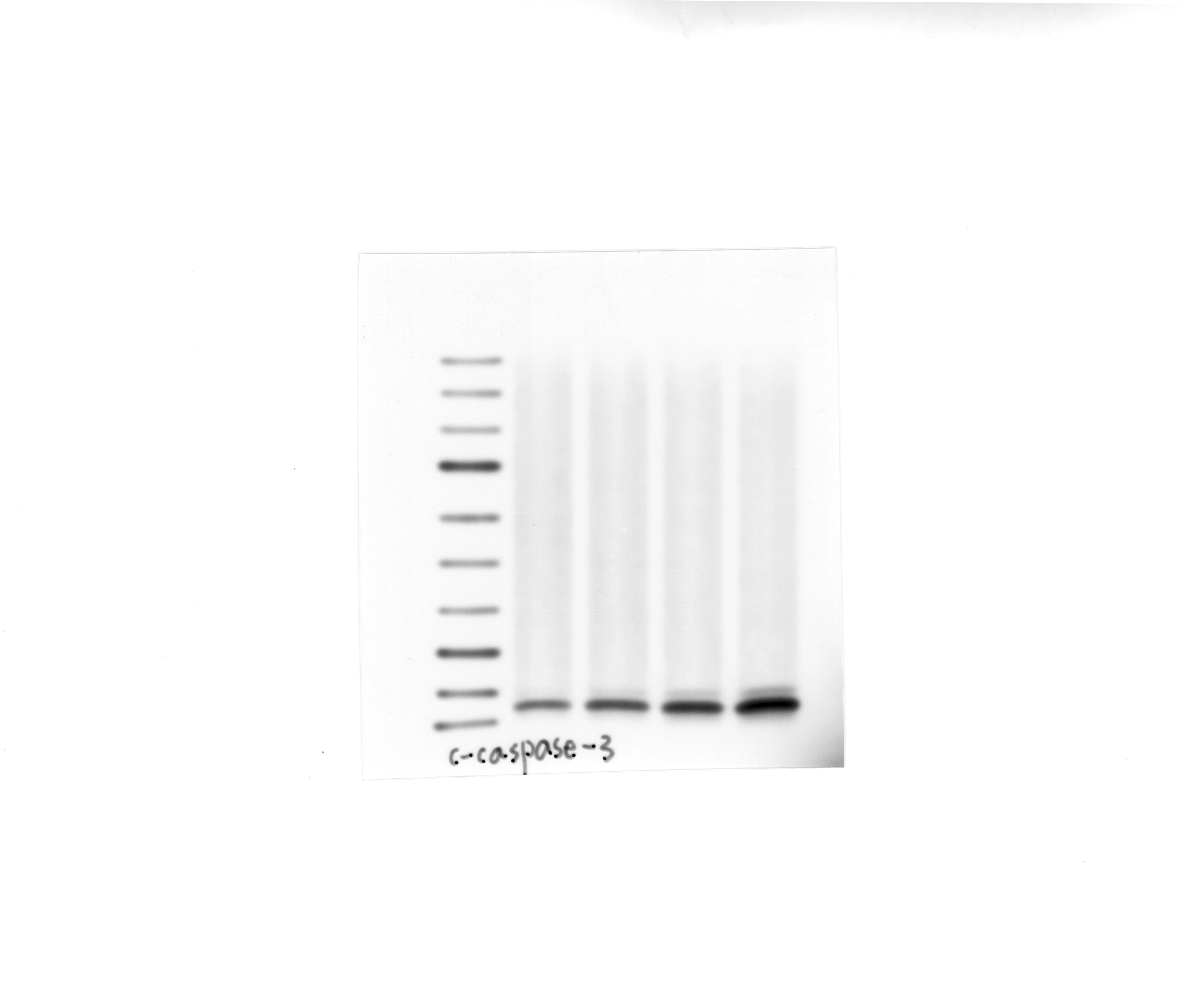

Supplement: Supplementary file 1 — Supplementary Information. [file 41598_2023_33792_MOESM1_ESM.zip › WB/fig 1E-SW480/cleaved caspase 3.tif]

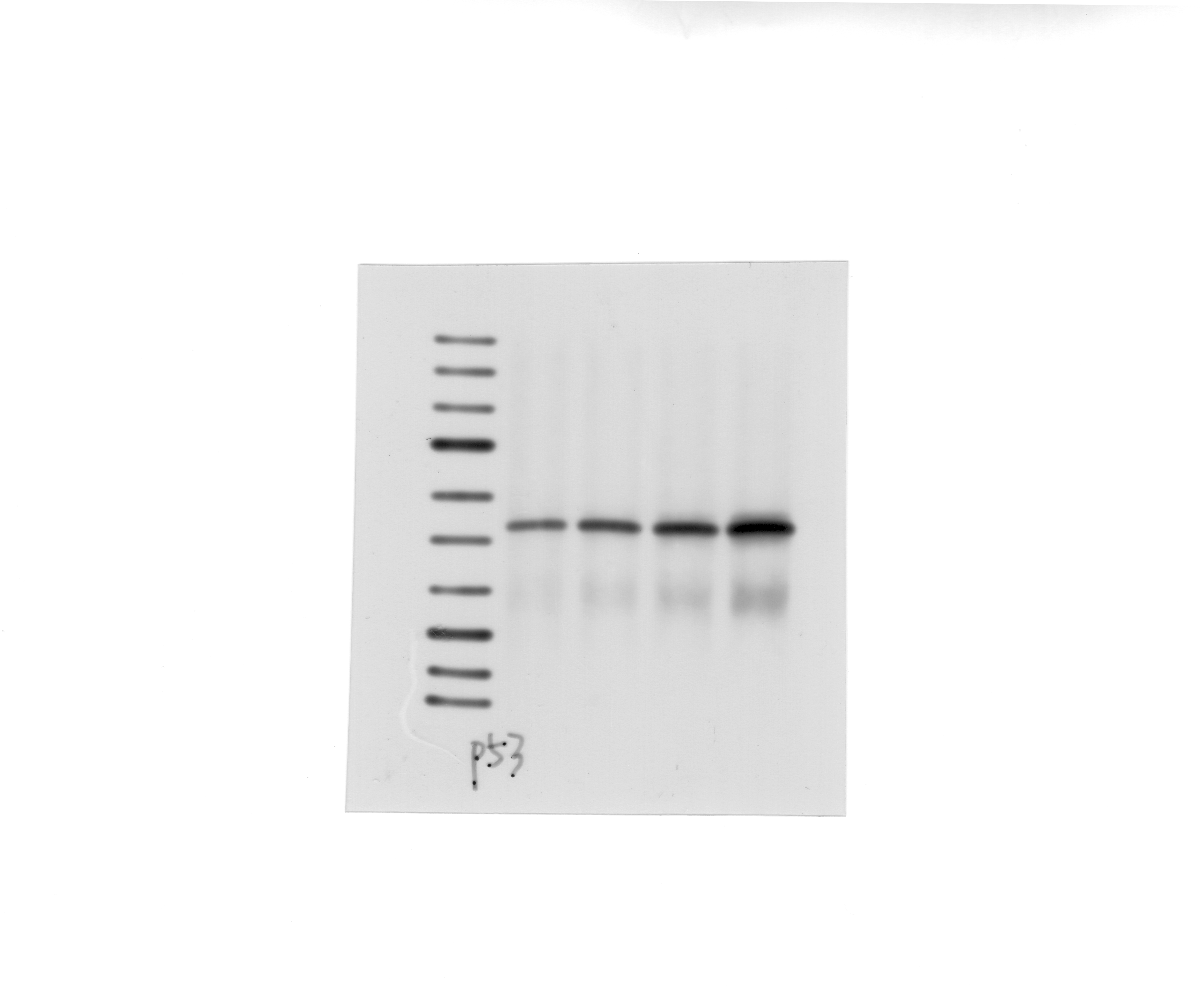

Supplement: Supplementary file 1 — Supplementary Information. [file 41598_2023_33792_MOESM1_ESM.zip › WB/fig 1E-SW480/p53.tif]

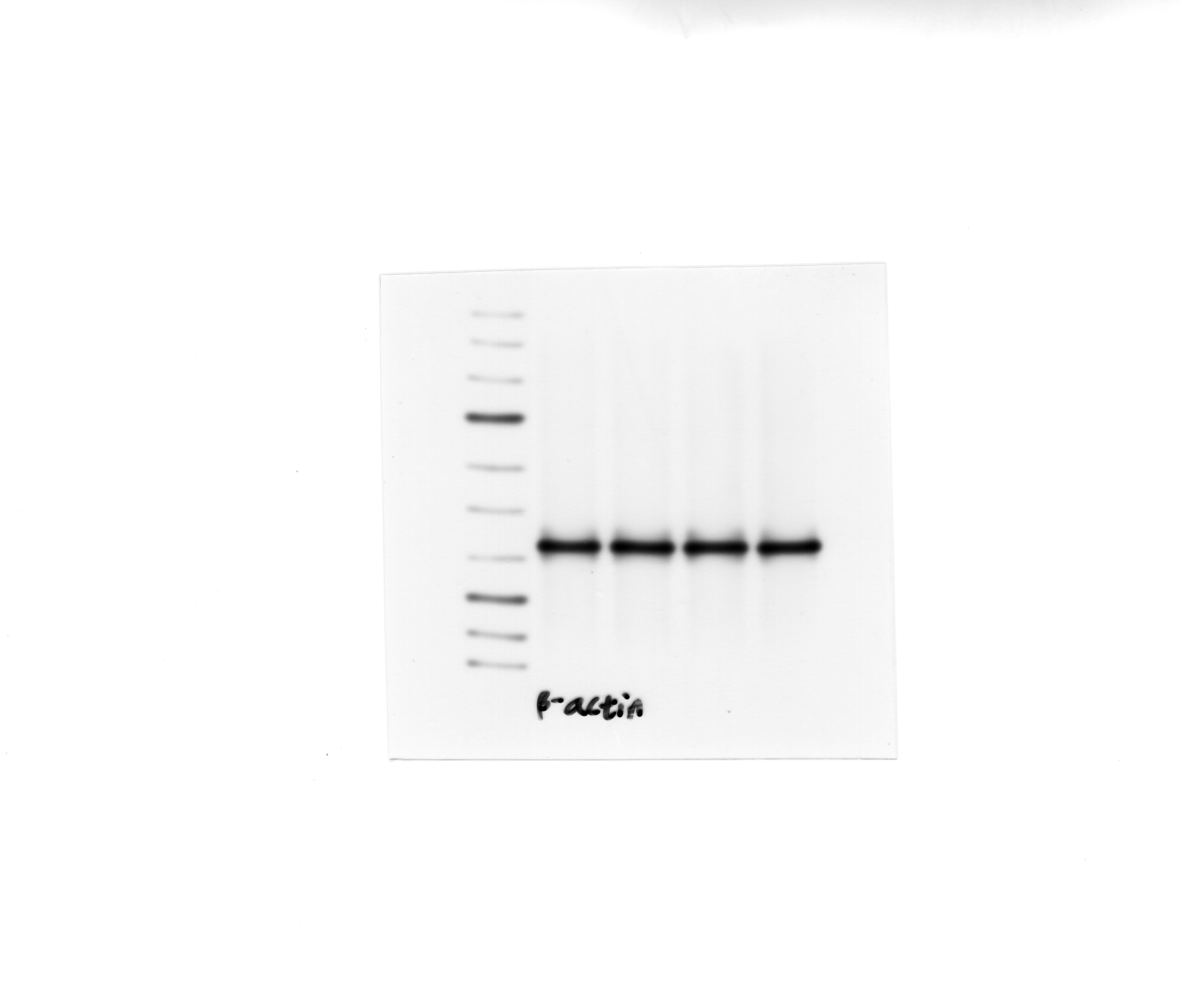

Supplement: Supplementary file 1 — Supplementary Information. [file 41598_2023_33792_MOESM1_ESM.zip › WB/fig 2D-HCT116/Actin.tif]

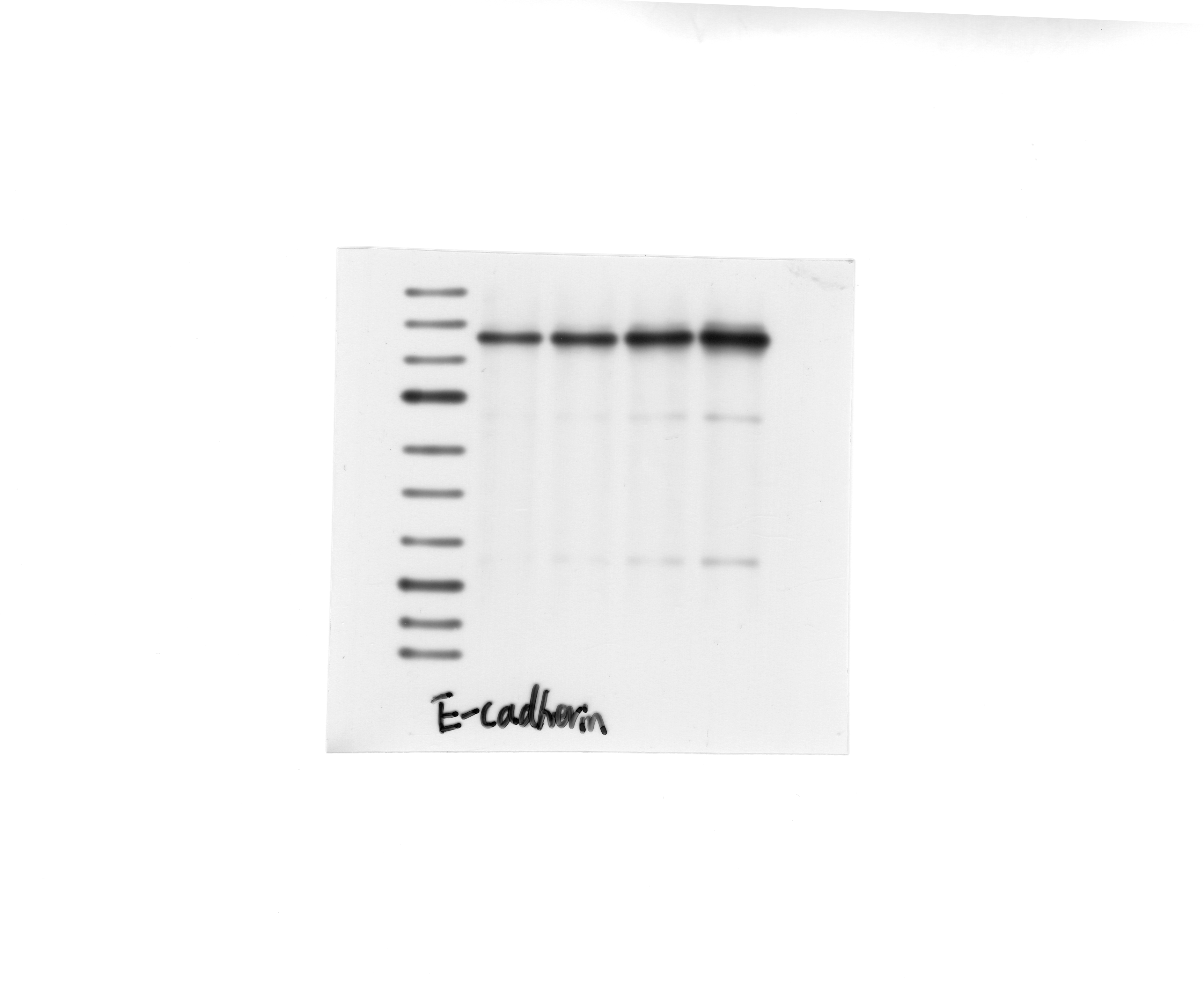

Supplement: Supplementary file 1 — Supplementary Information. [file 41598_2023_33792_MOESM1_ESM.zip › WB/fig 2D-HCT116/E-cadherin.tif]

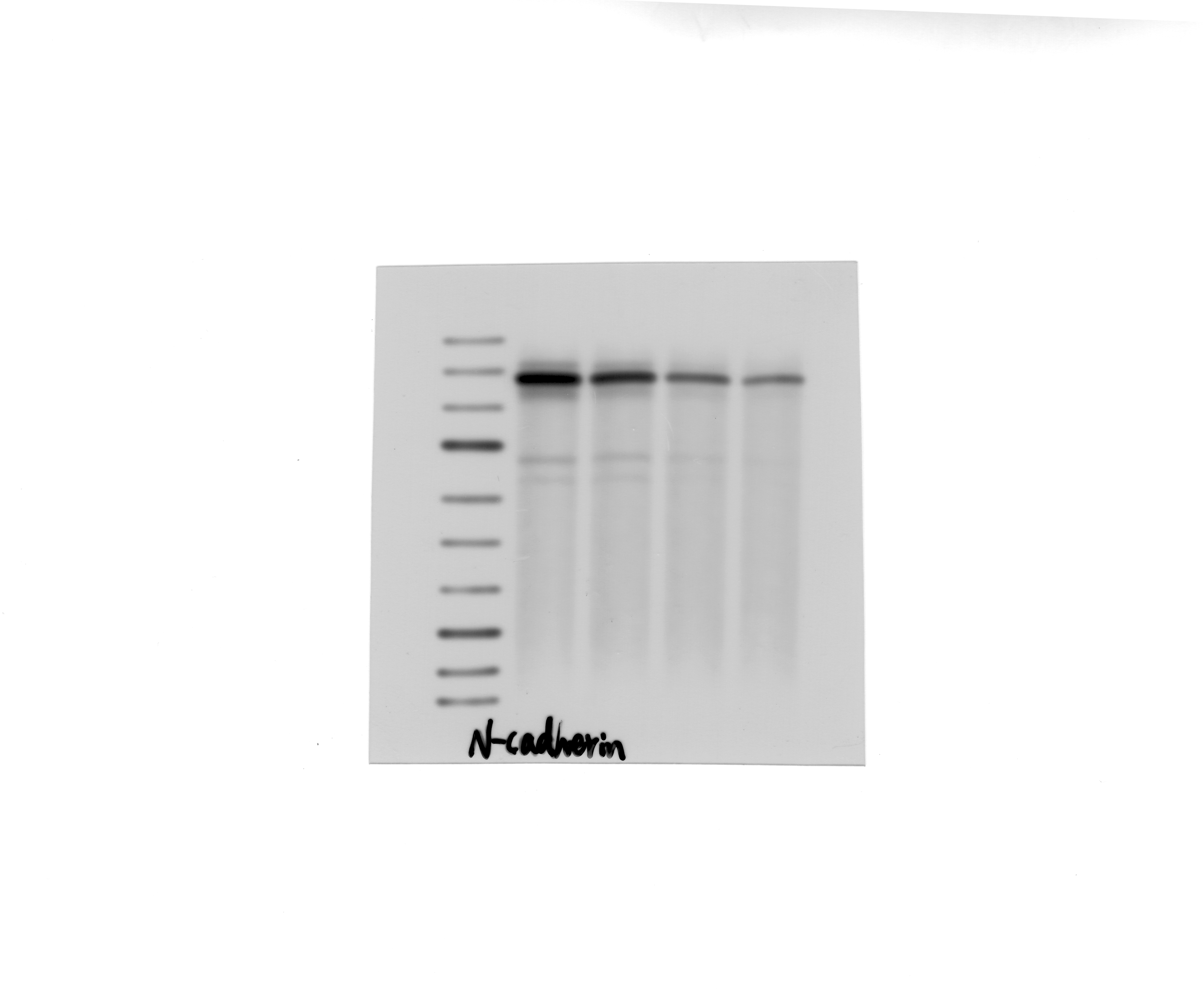

Supplement: Supplementary file 1 — Supplementary Information. [file 41598_2023_33792_MOESM1_ESM.zip › WB/fig 2D-HCT116/N-cadherin.tif]

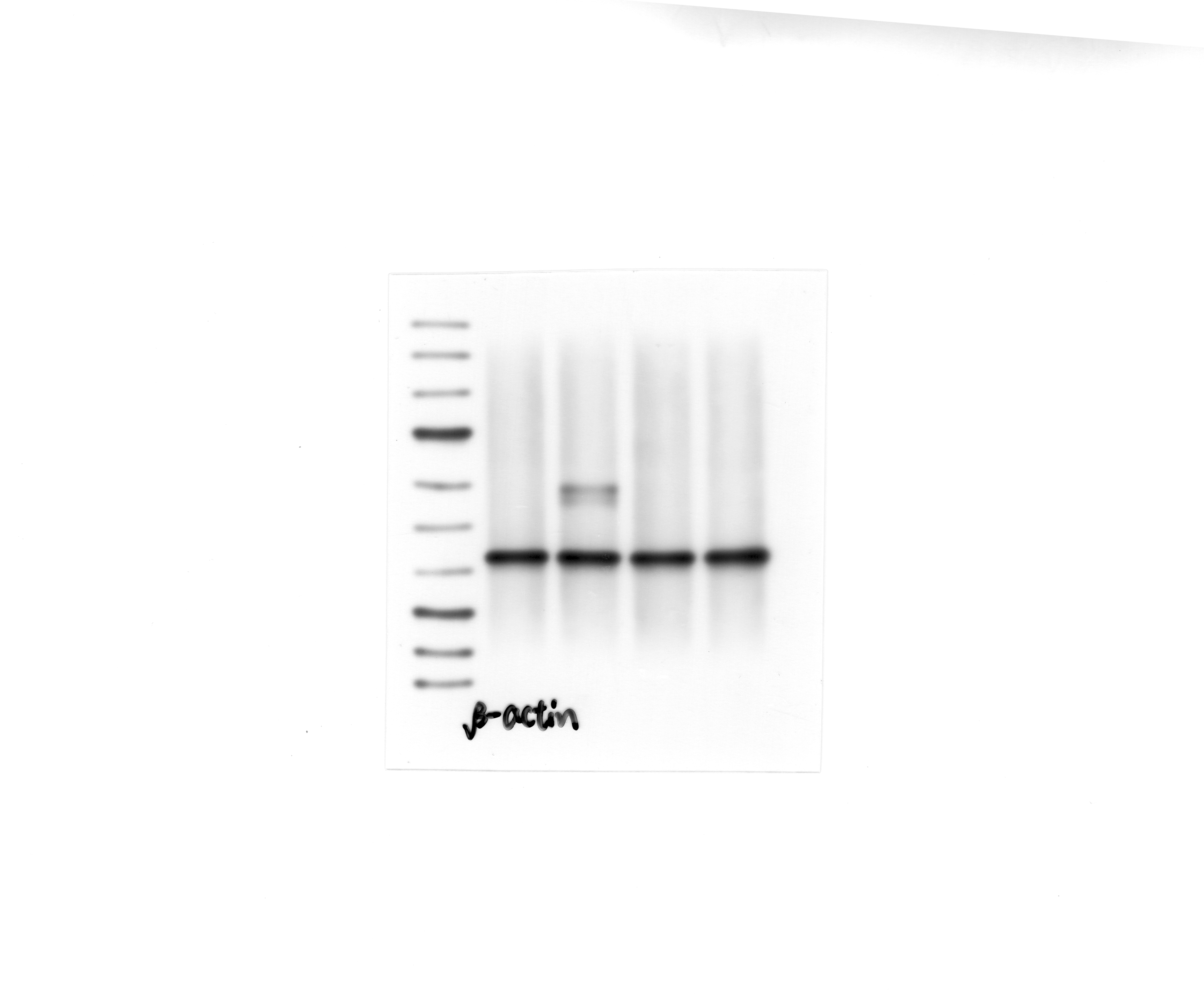

Supplement: Supplementary file 1 — Supplementary Information. [file 41598_2023_33792_MOESM1_ESM.zip › WB/fig 2D-SW480/Actin.tif]

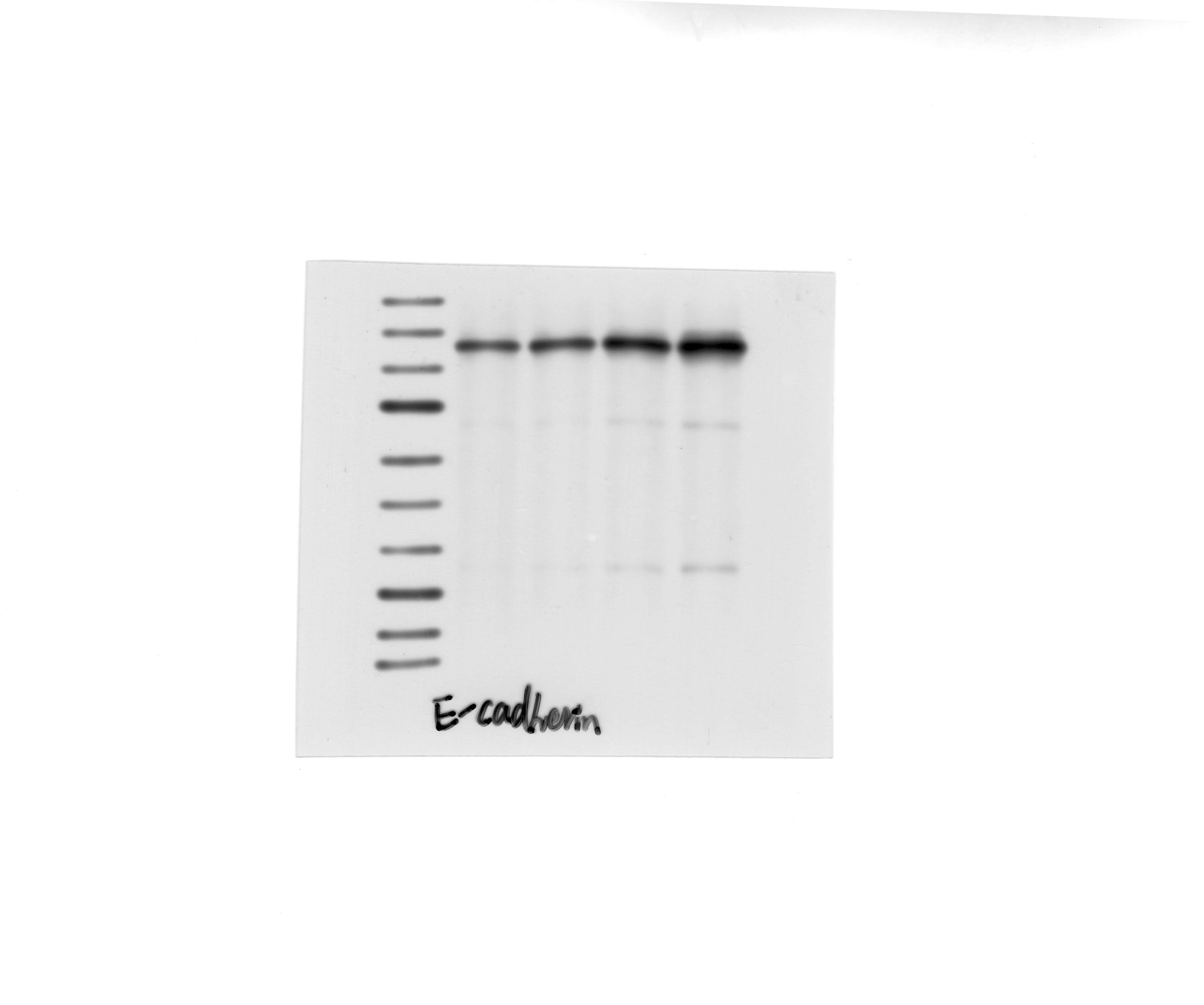

Supplement: Supplementary file 1 — Supplementary Information. [file 41598_2023_33792_MOESM1_ESM.zip › WB/fig 2D-SW480/E-cadherin.tif]

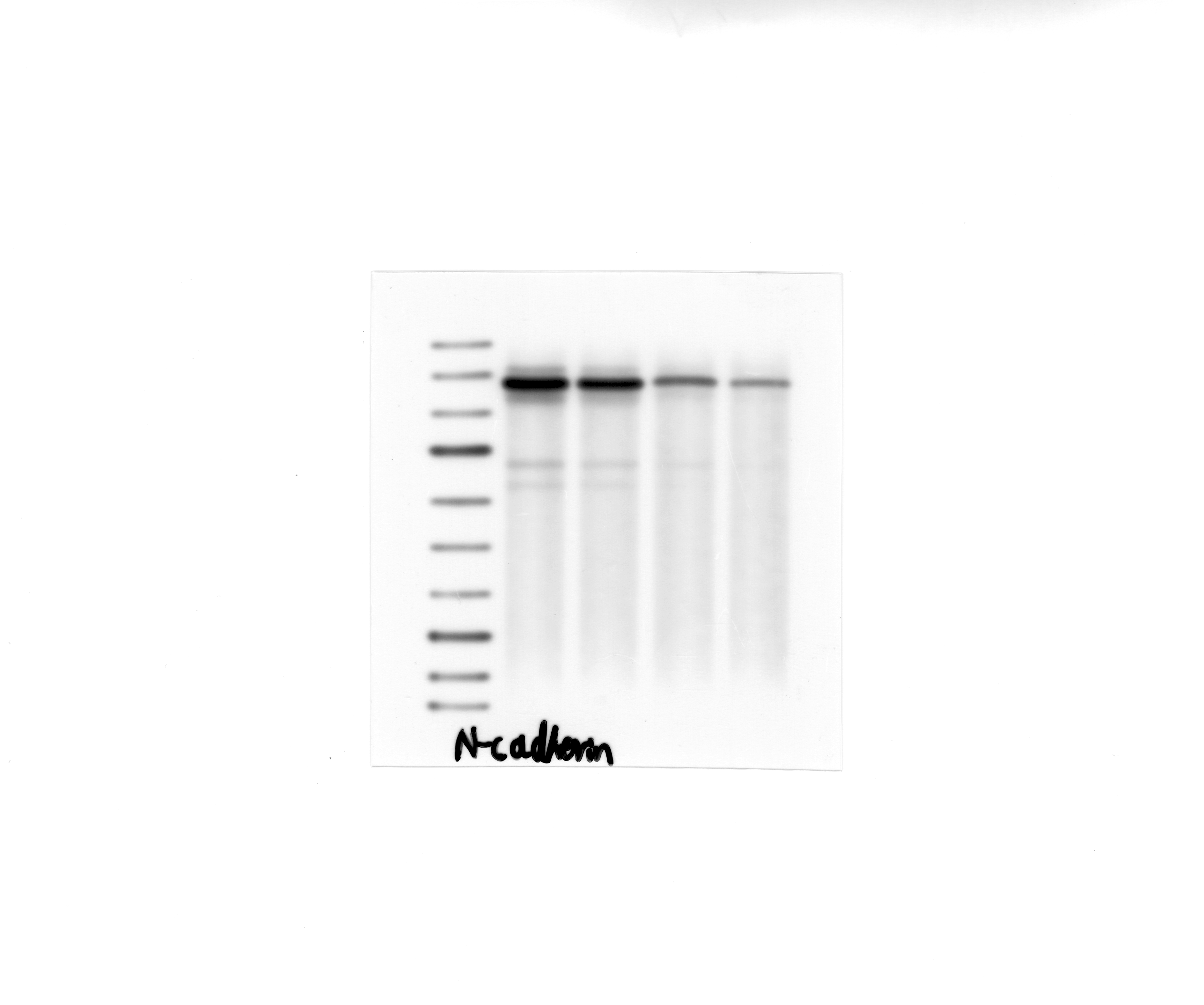

Supplement: Supplementary file 1 — Supplementary Information. [file 41598_2023_33792_MOESM1_ESM.zip › WB/fig 2D-SW480/N-cadherin.tif]

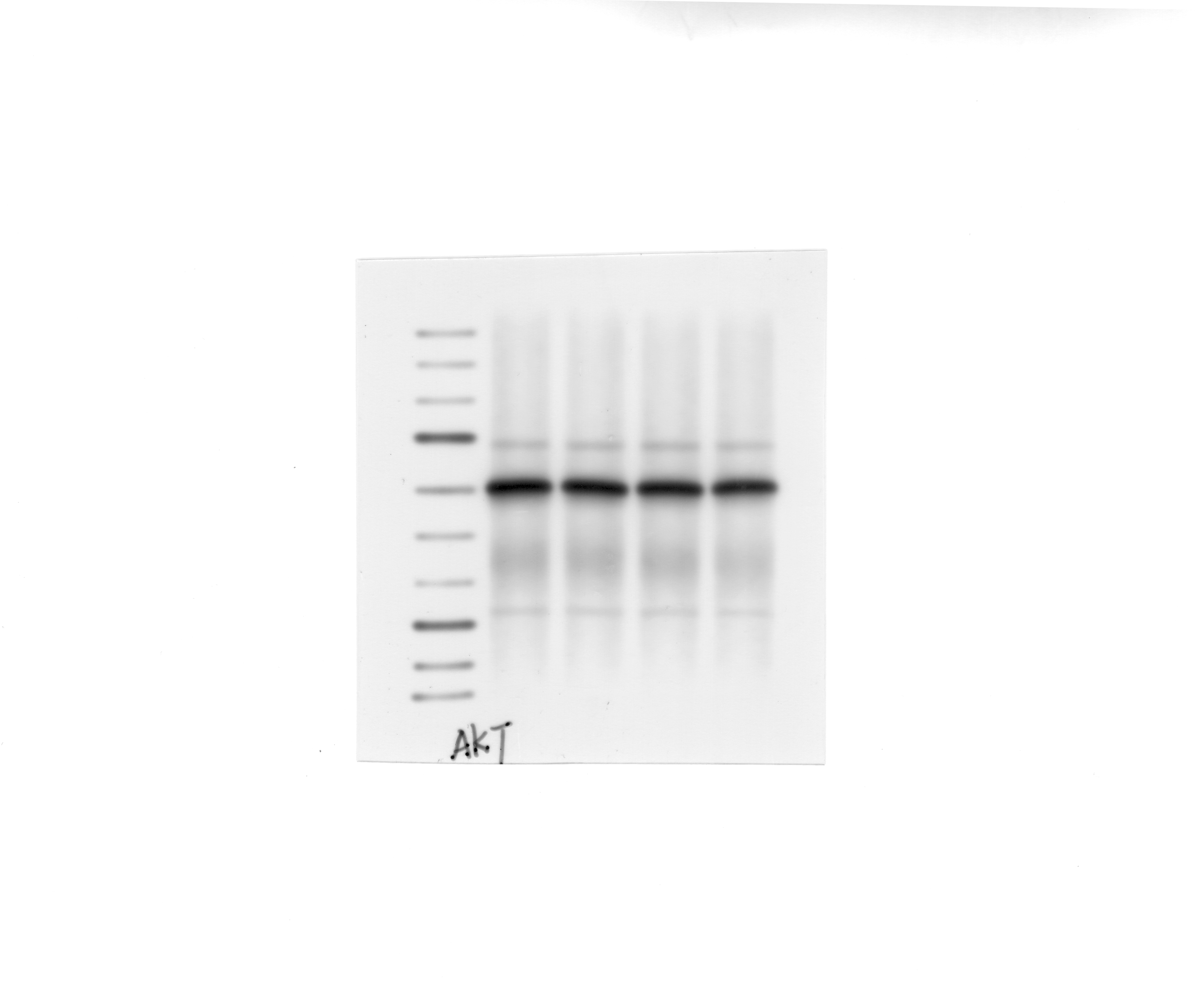

Supplement: Supplementary file 1 — Supplementary Information. [file 41598_2023_33792_MOESM1_ESM.zip › WB/fig 3A-HCT116/AKT.tif]

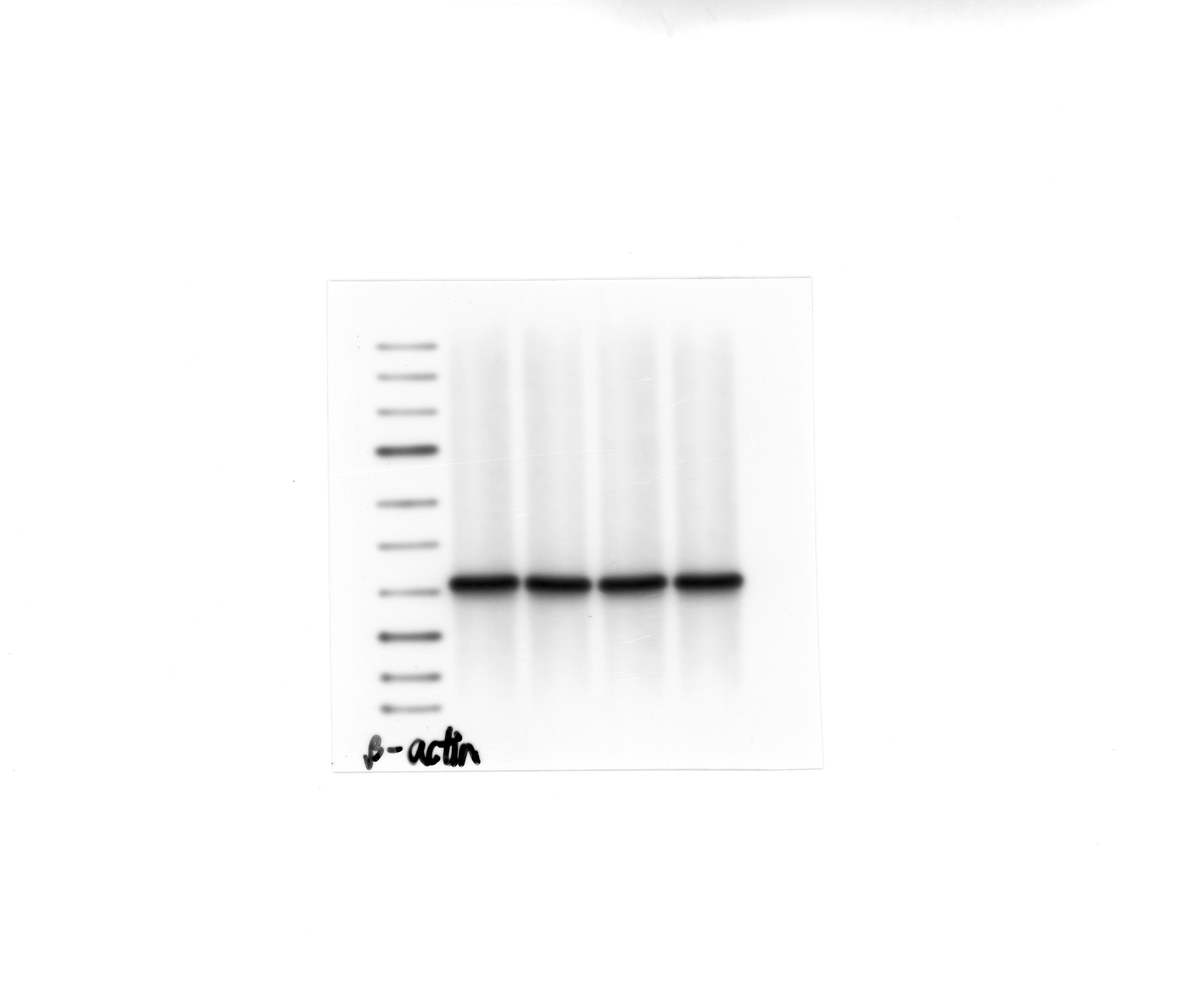

Supplement: Supplementary file 1 — Supplementary Information. [file 41598_2023_33792_MOESM1_ESM.zip › WB/fig 3A-HCT116/Actin.tif]

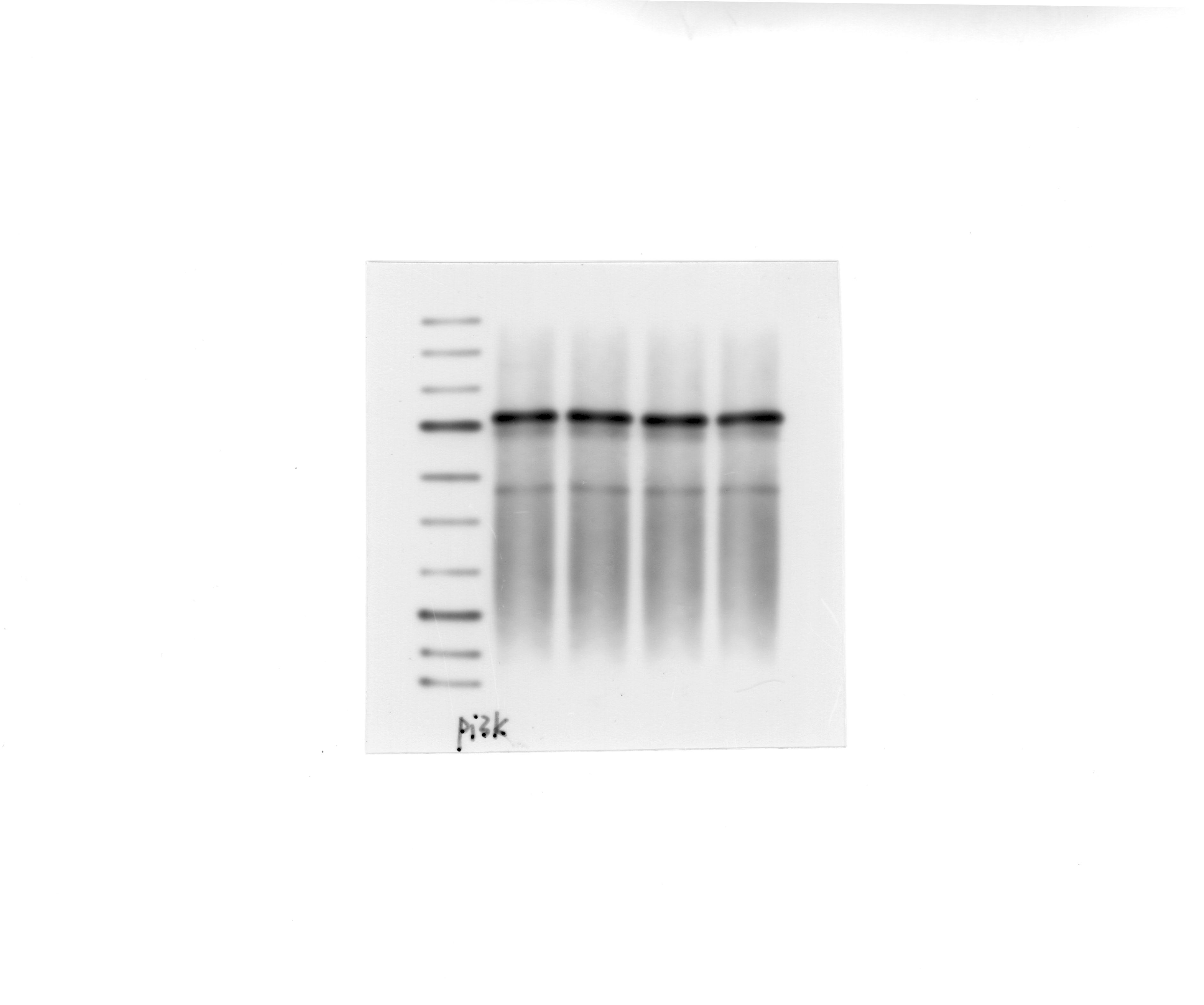

Supplement: Supplementary file 1 — Supplementary Information. [file 41598_2023_33792_MOESM1_ESM.zip › WB/fig 3A-HCT116/PI3K.tif]

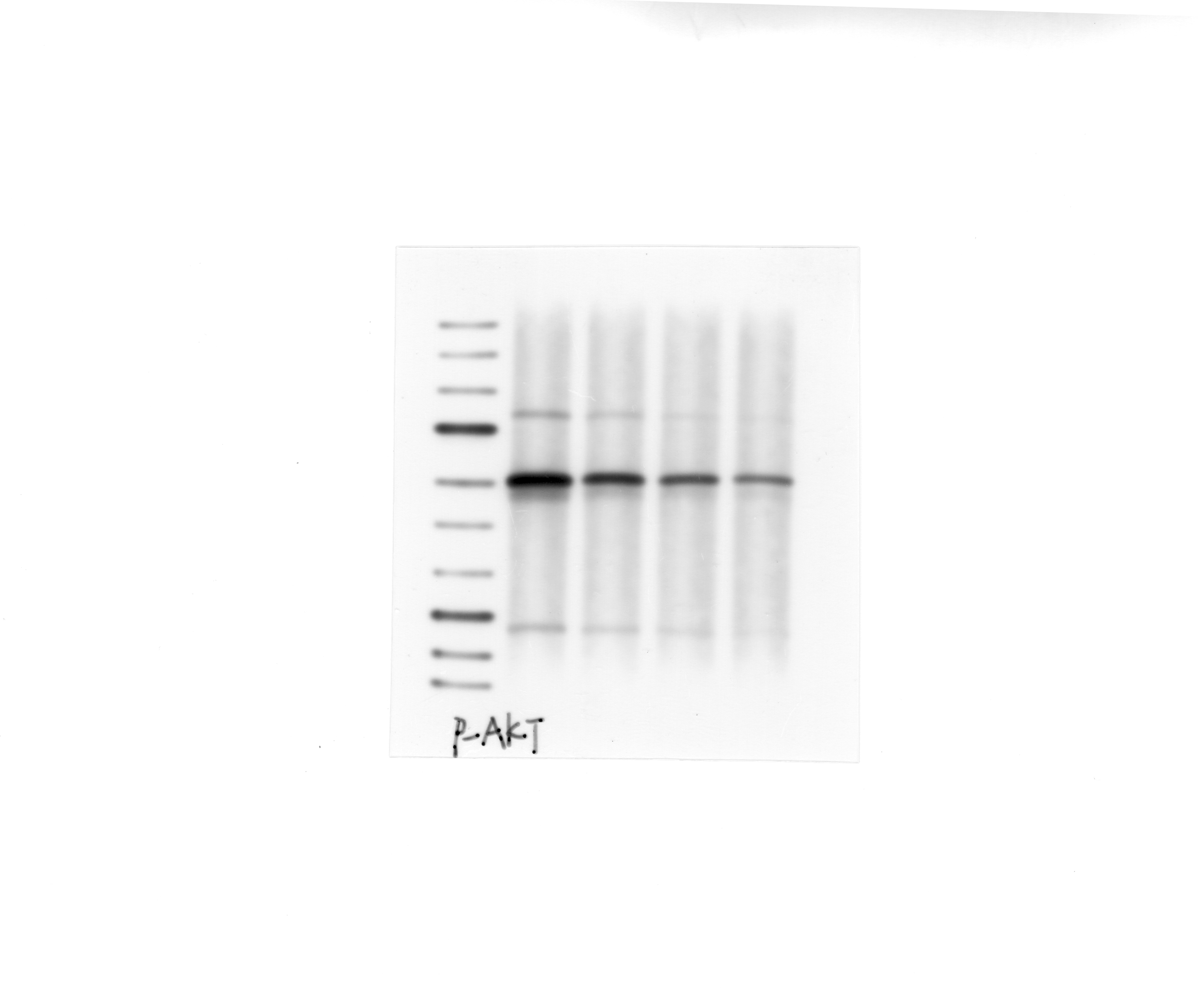

Supplement: Supplementary file 1 — Supplementary Information. [file 41598_2023_33792_MOESM1_ESM.zip › WB/fig 3A-HCT116/p-AKT.tif]

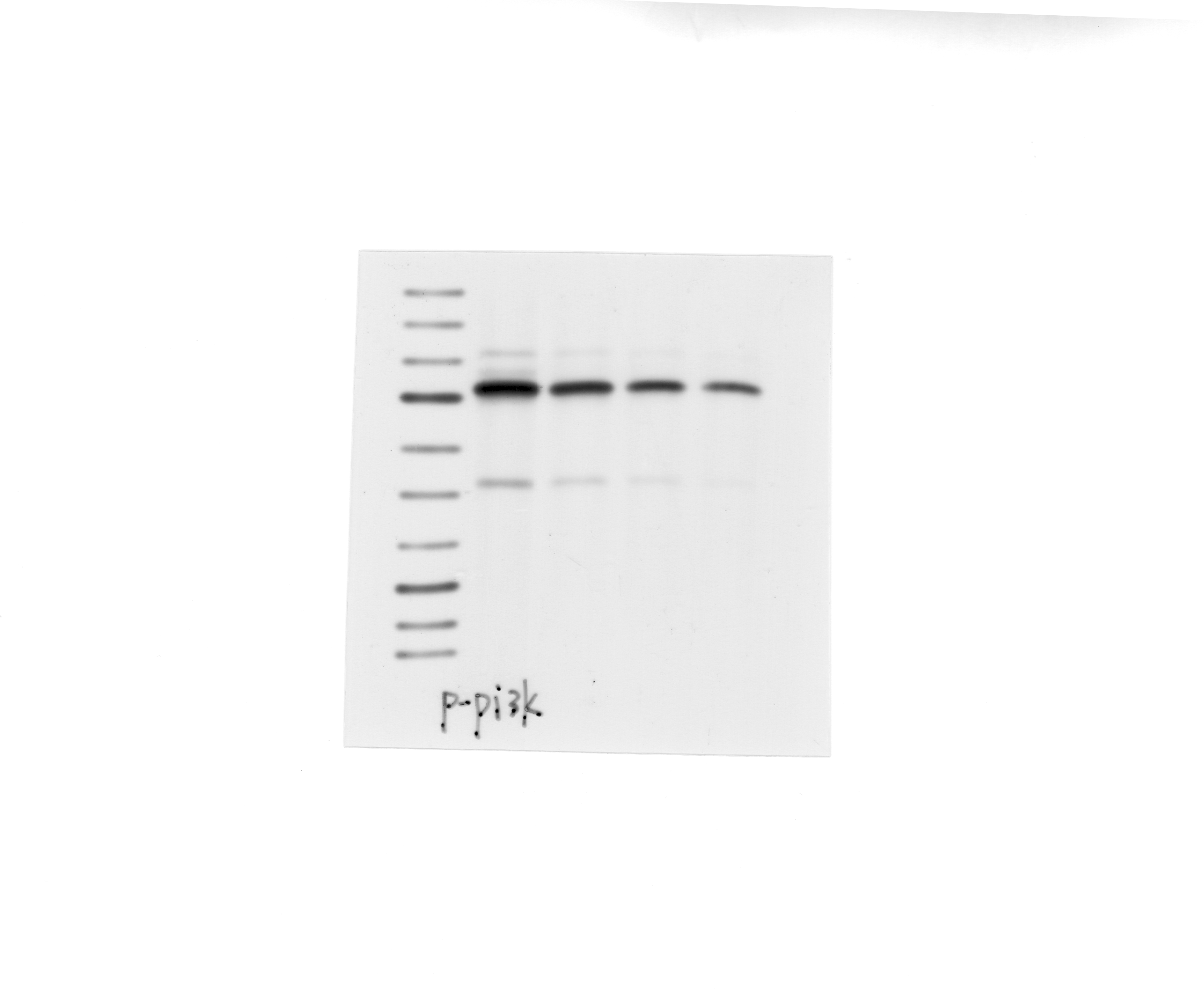

Supplement: Supplementary file 1 — Supplementary Information. [file 41598_2023_33792_MOESM1_ESM.zip › WB/fig 3A-HCT116/p-PI3K.tif]

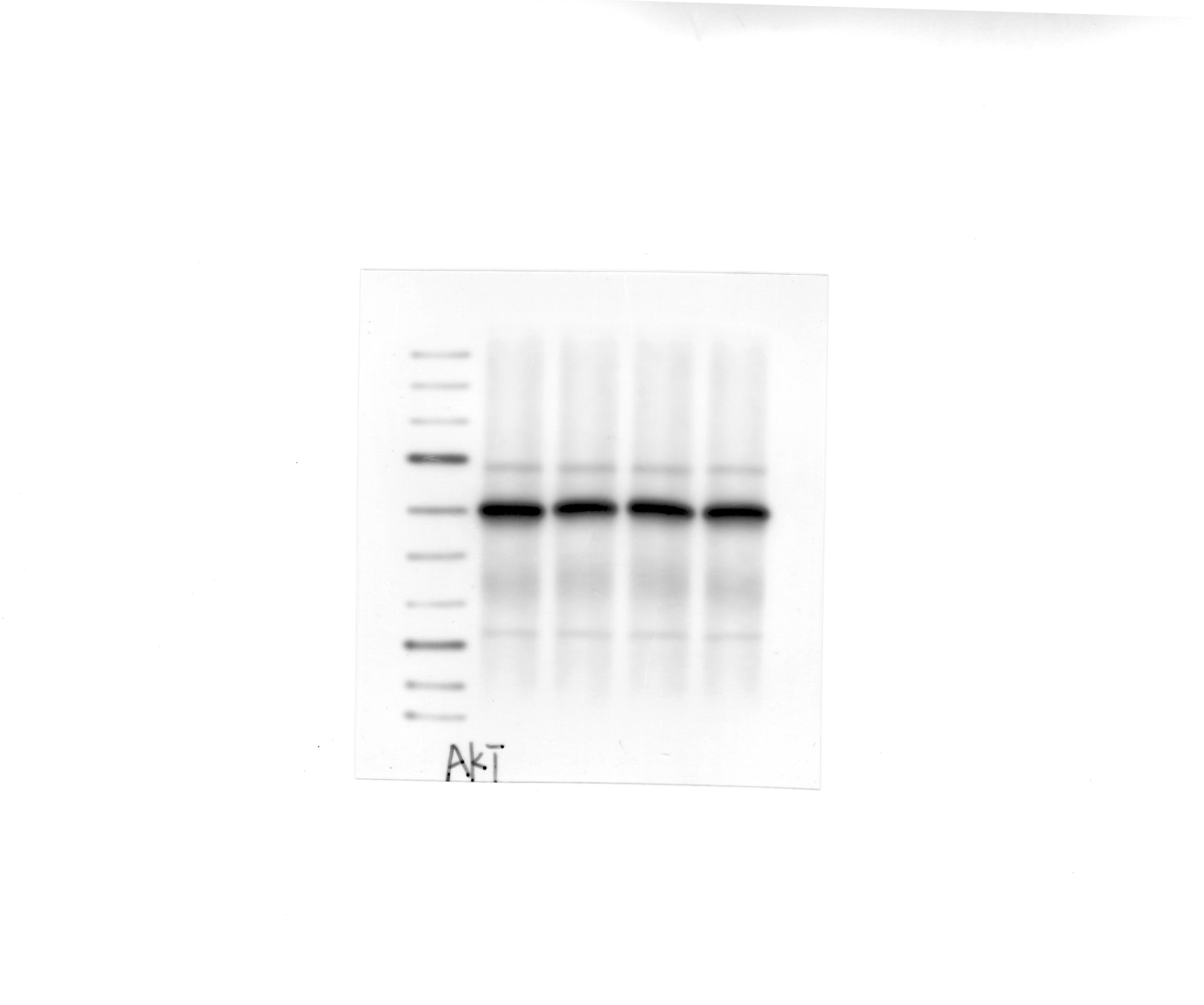

Supplement: Supplementary file 1 — Supplementary Information. [file 41598_2023_33792_MOESM1_ESM.zip › WB/fig 3A-SW480/AKT.tif]

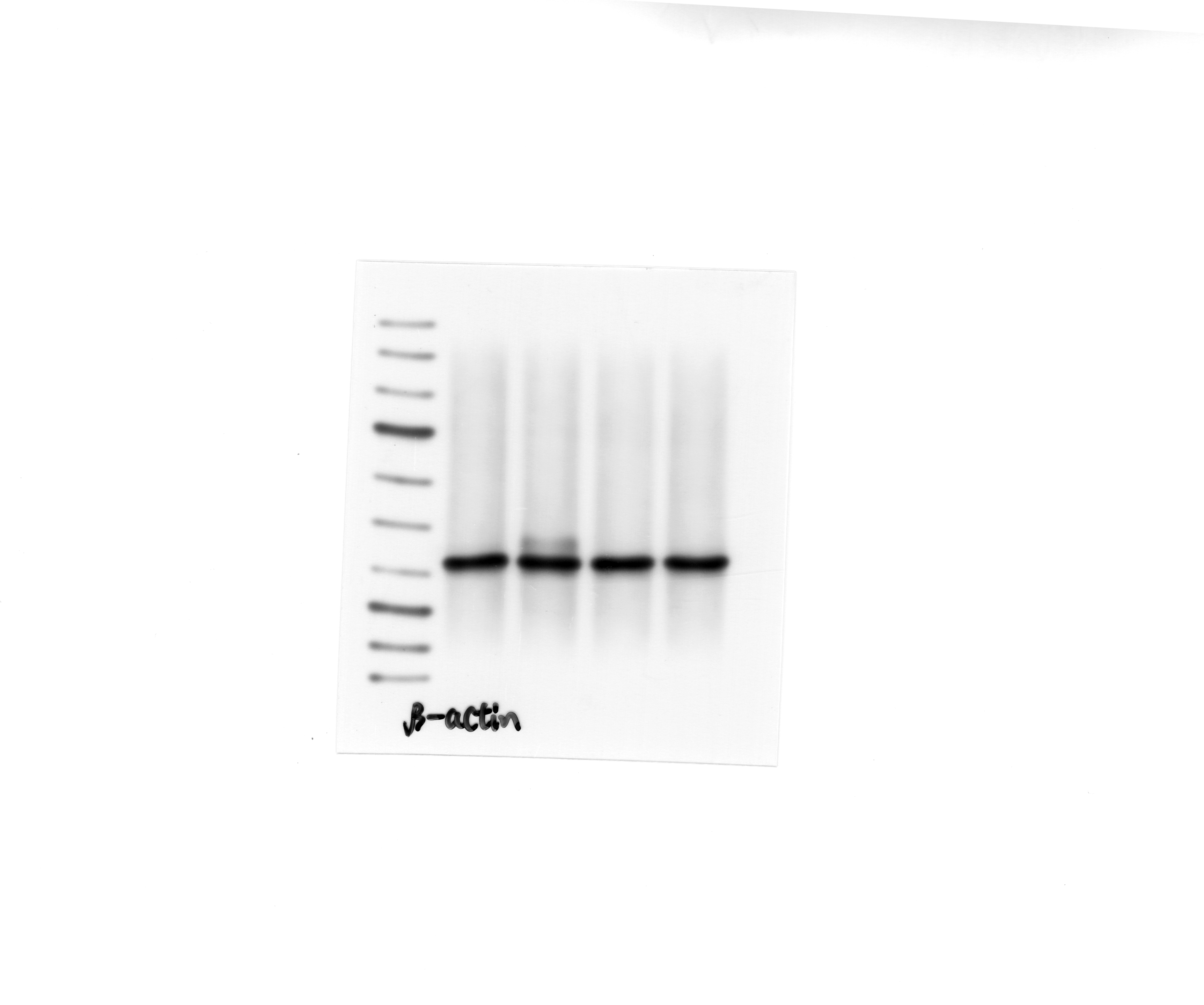

Supplement: Supplementary file 1 — Supplementary Information. [file 41598_2023_33792_MOESM1_ESM.zip › WB/fig 3A-SW480/Actin.tif]

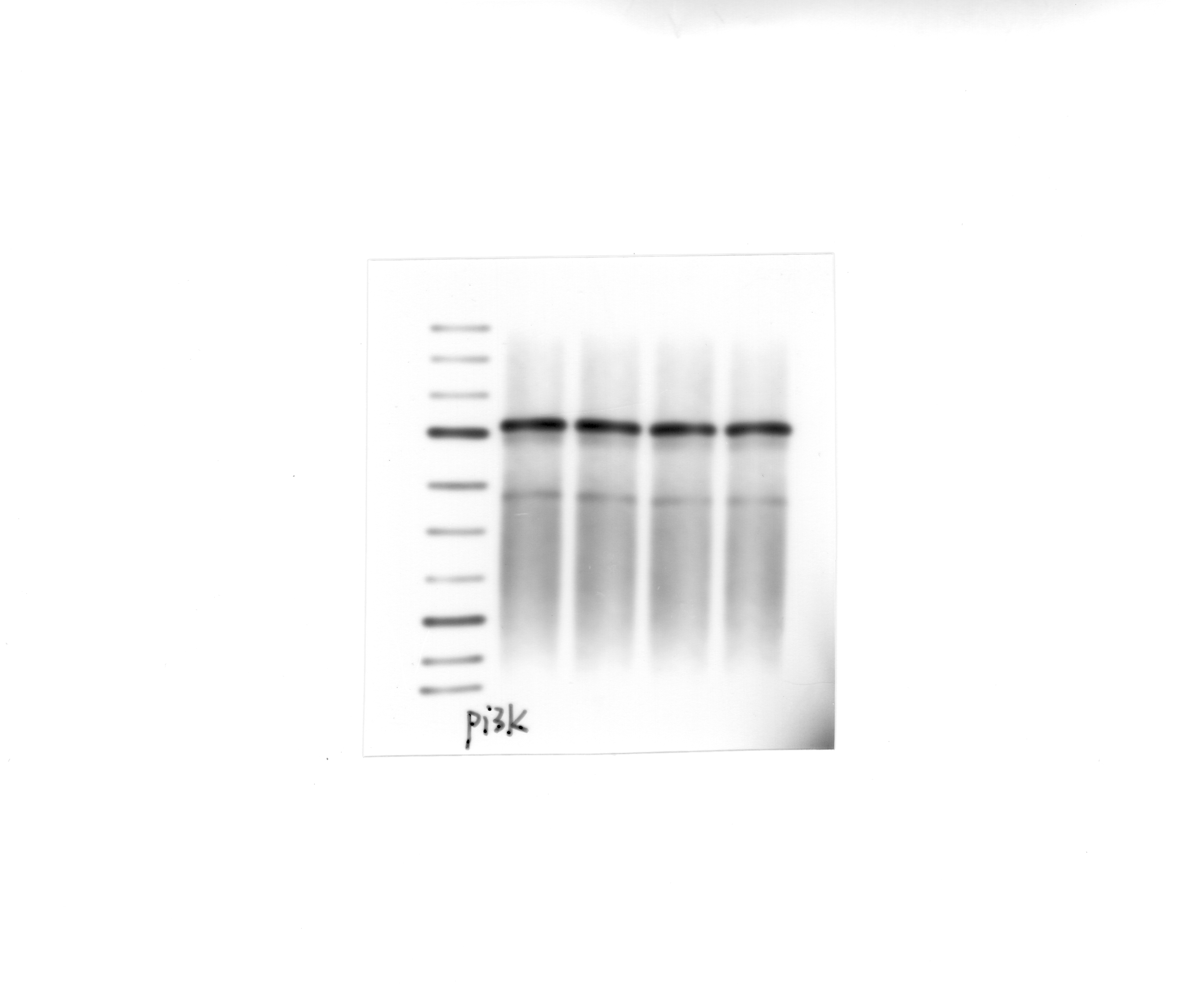

Supplement: Supplementary file 1 — Supplementary Information. [file 41598_2023_33792_MOESM1_ESM.zip › WB/fig 3A-SW480/PI3K.tif]

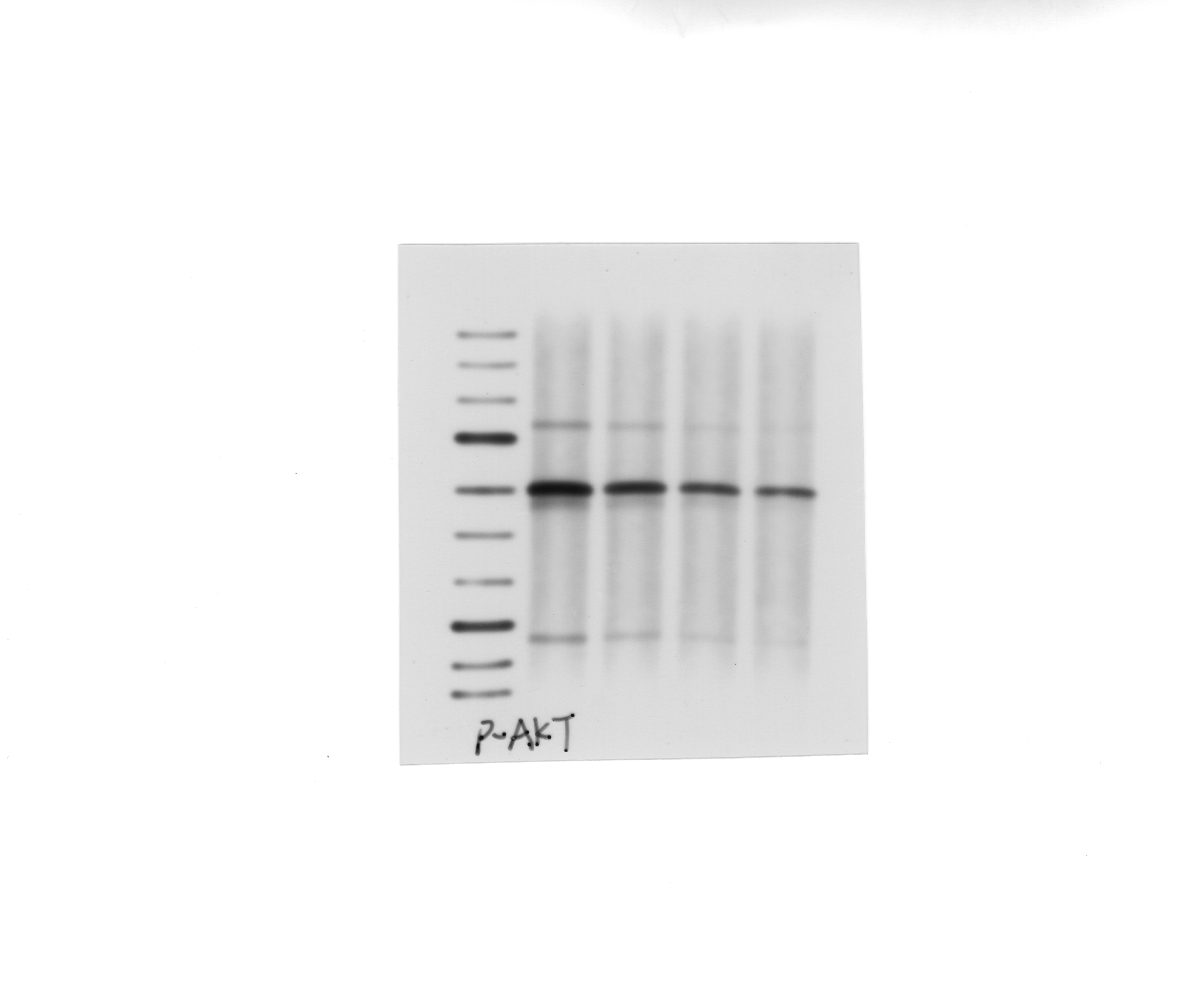

Supplement: Supplementary file 1 — Supplementary Information. [file 41598_2023_33792_MOESM1_ESM.zip › WB/fig 3A-SW480/p-AKT.tif]

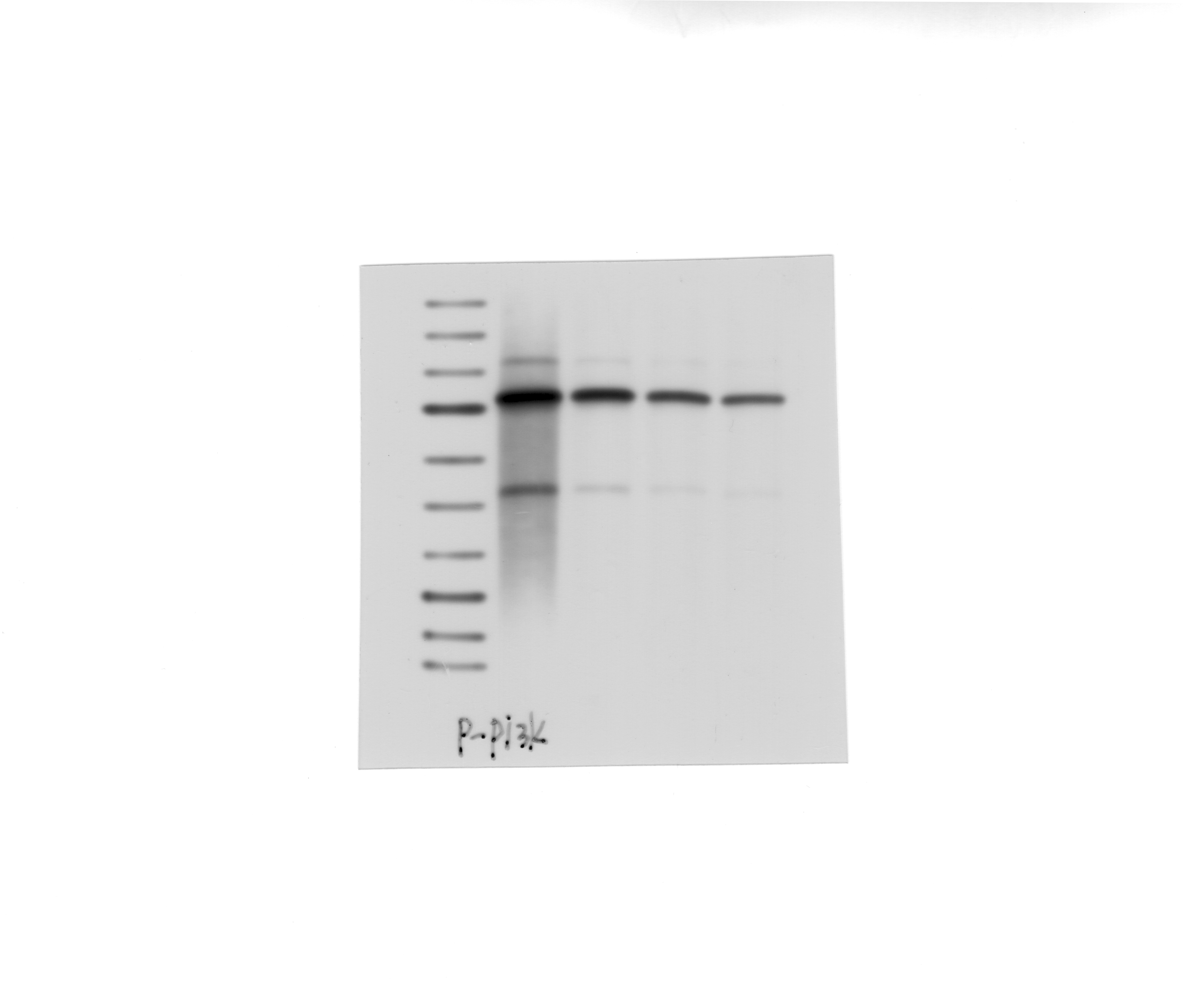

Supplement: Supplementary file 1 — Supplementary Information. [file 41598_2023_33792_MOESM1_ESM.zip › WB/fig 3A-SW480/p-PI3K.tif]

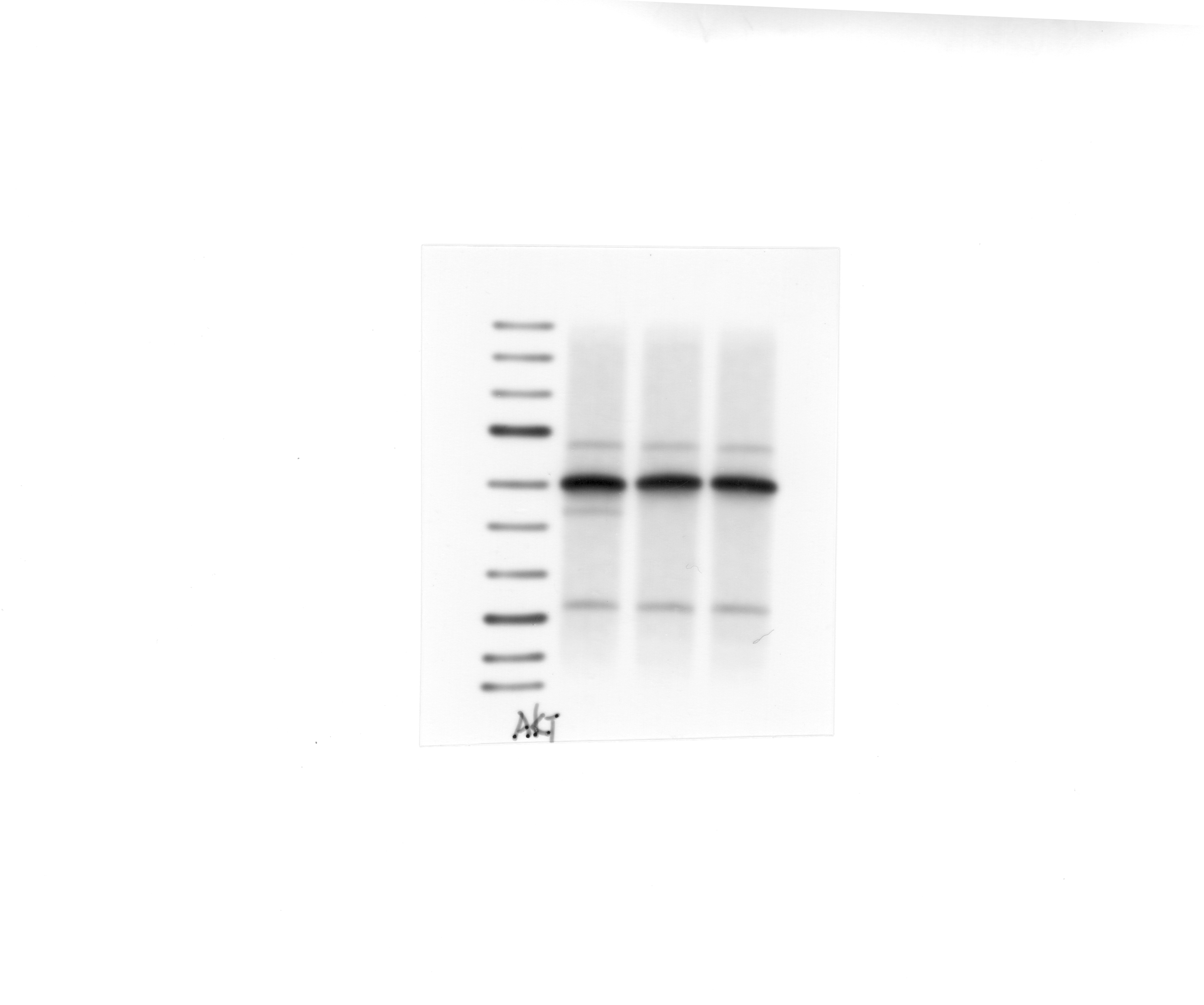

Supplement: Supplementary file 1 — Supplementary Information. [file 41598_2023_33792_MOESM1_ESM.zip › WB/fig 3B-HCT116/AKT.tif]

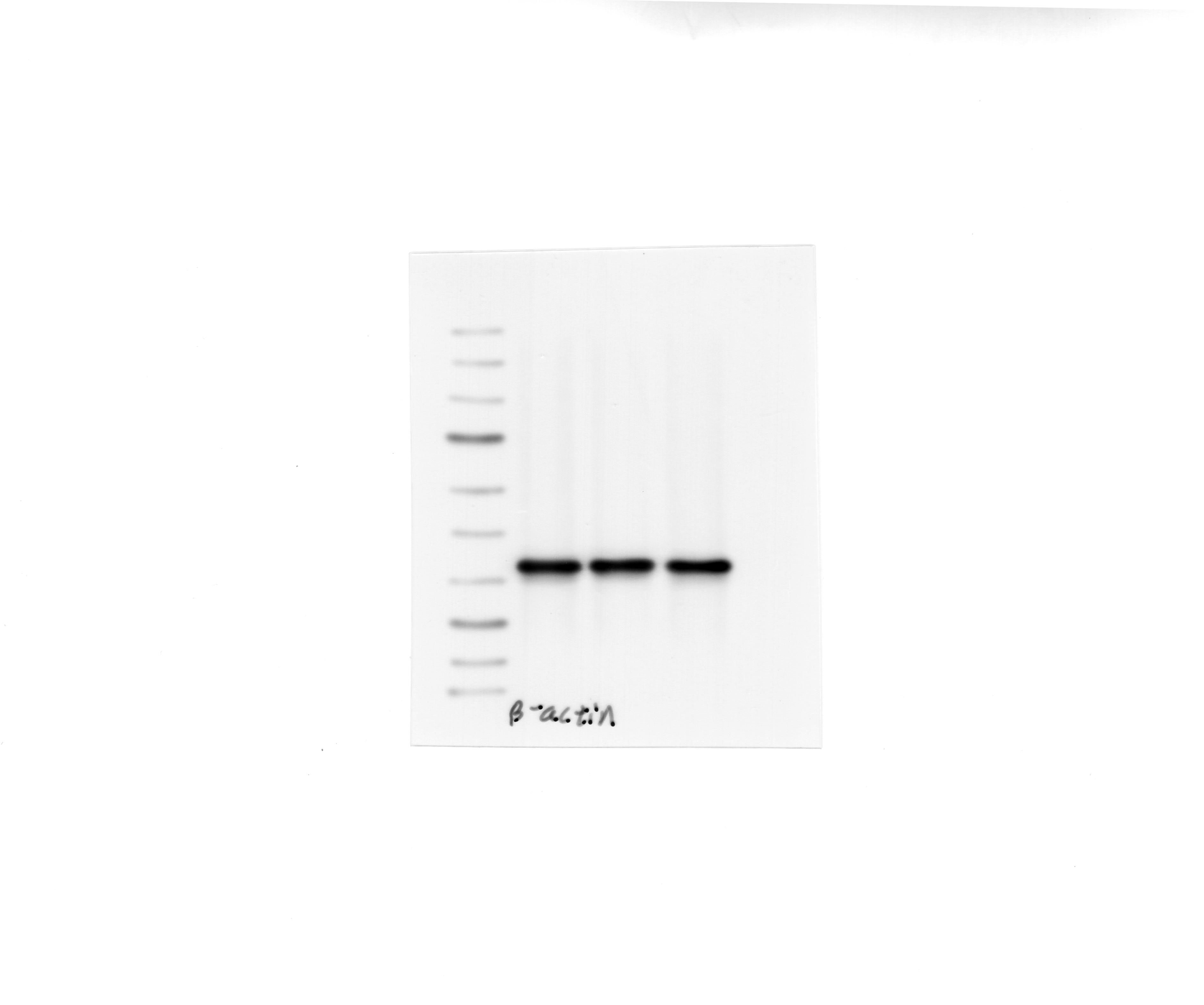

Supplement: Supplementary file 1 — Supplementary Information. [file 41598_2023_33792_MOESM1_ESM.zip › WB/fig 3B-HCT116/Actin.tif]

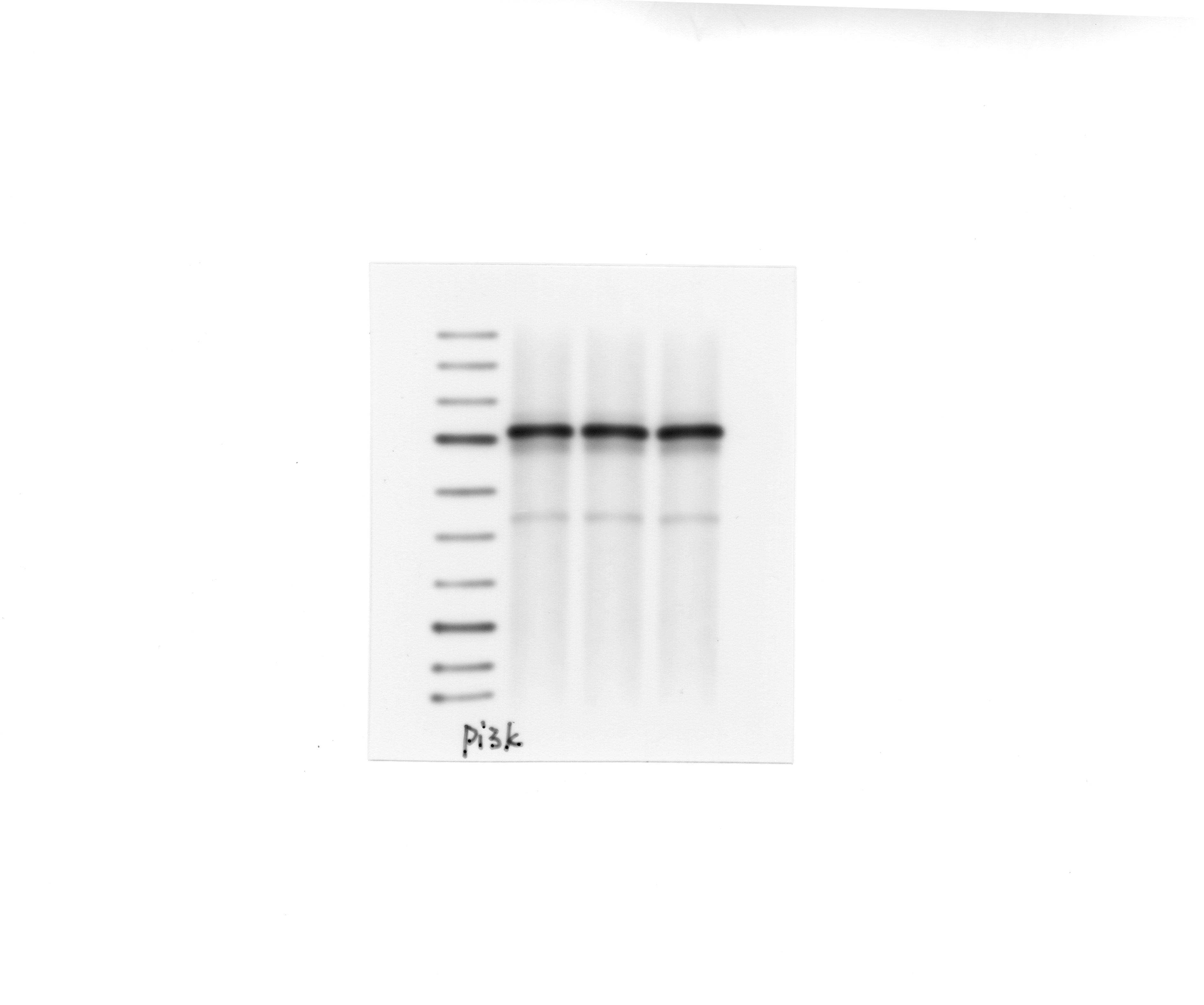

Supplement: Supplementary file 1 — Supplementary Information. [file 41598_2023_33792_MOESM1_ESM.zip › WB/fig 3B-HCT116/PI3K.tif]

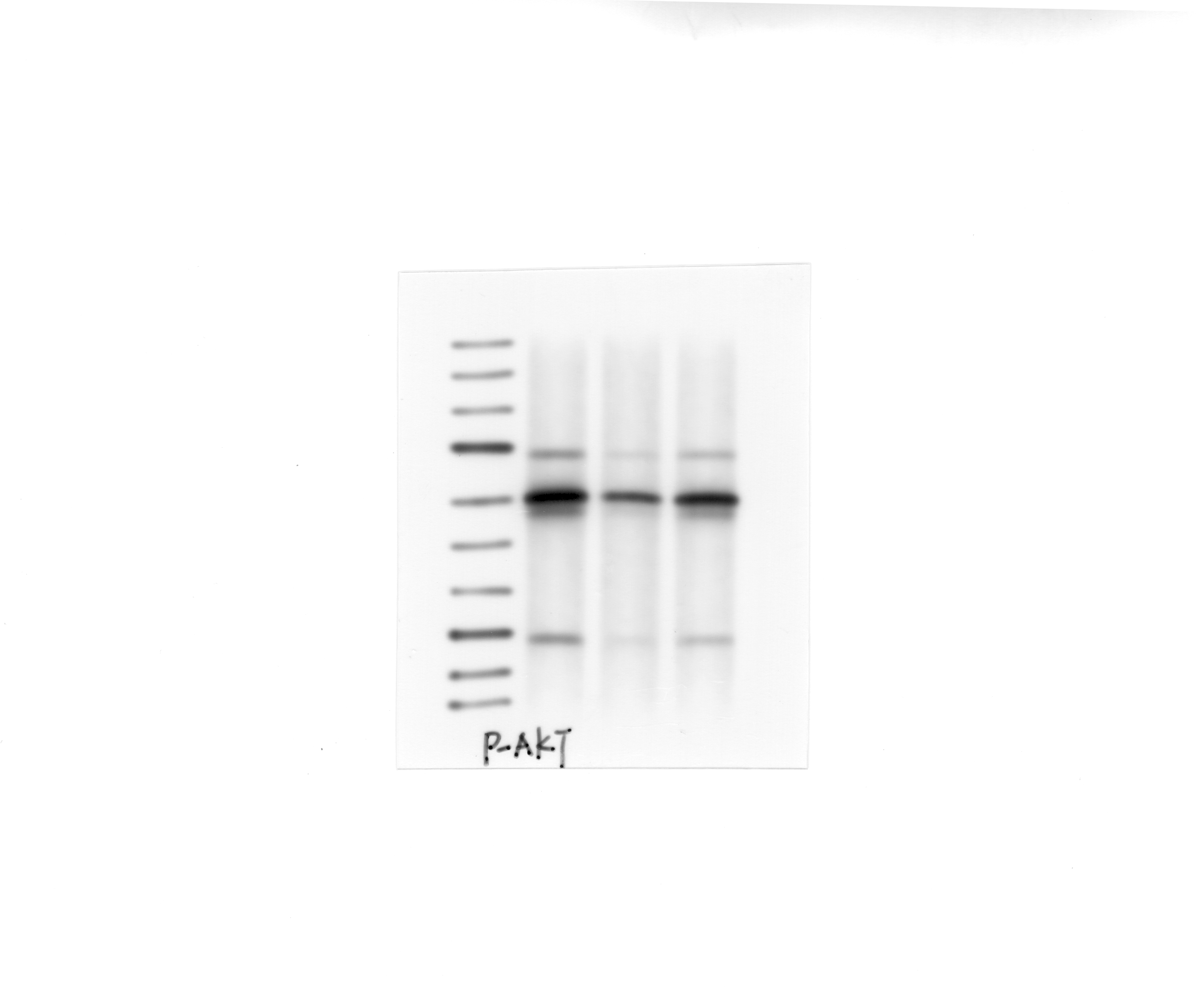

Supplement: Supplementary file 1 — Supplementary Information. [file 41598_2023_33792_MOESM1_ESM.zip › WB/fig 3B-HCT116/p-AKT.tif]

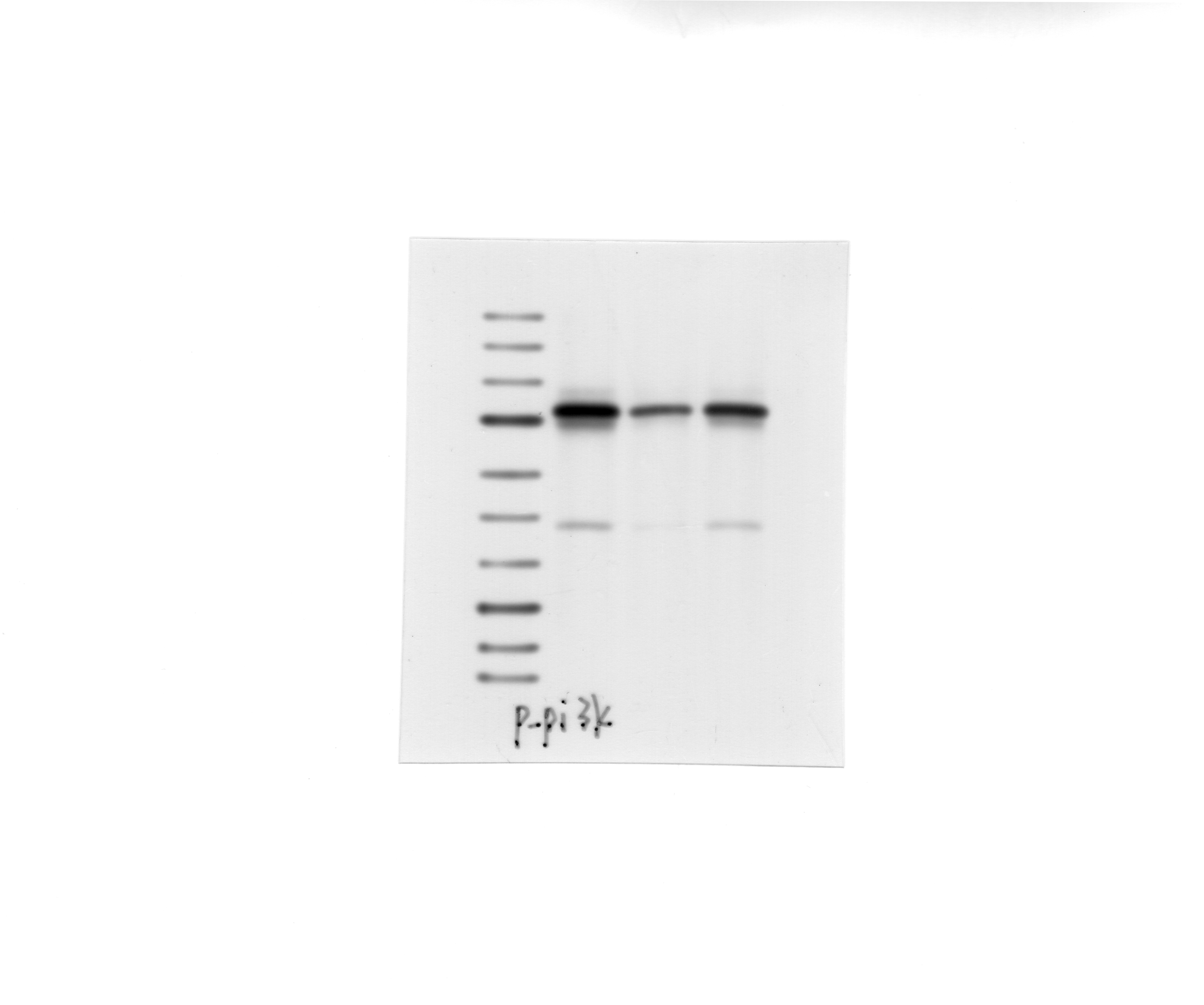

Supplement: Supplementary file 1 — Supplementary Information. [file 41598_2023_33792_MOESM1_ESM.zip › WB/fig 3B-HCT116/p-PI3K.tif]

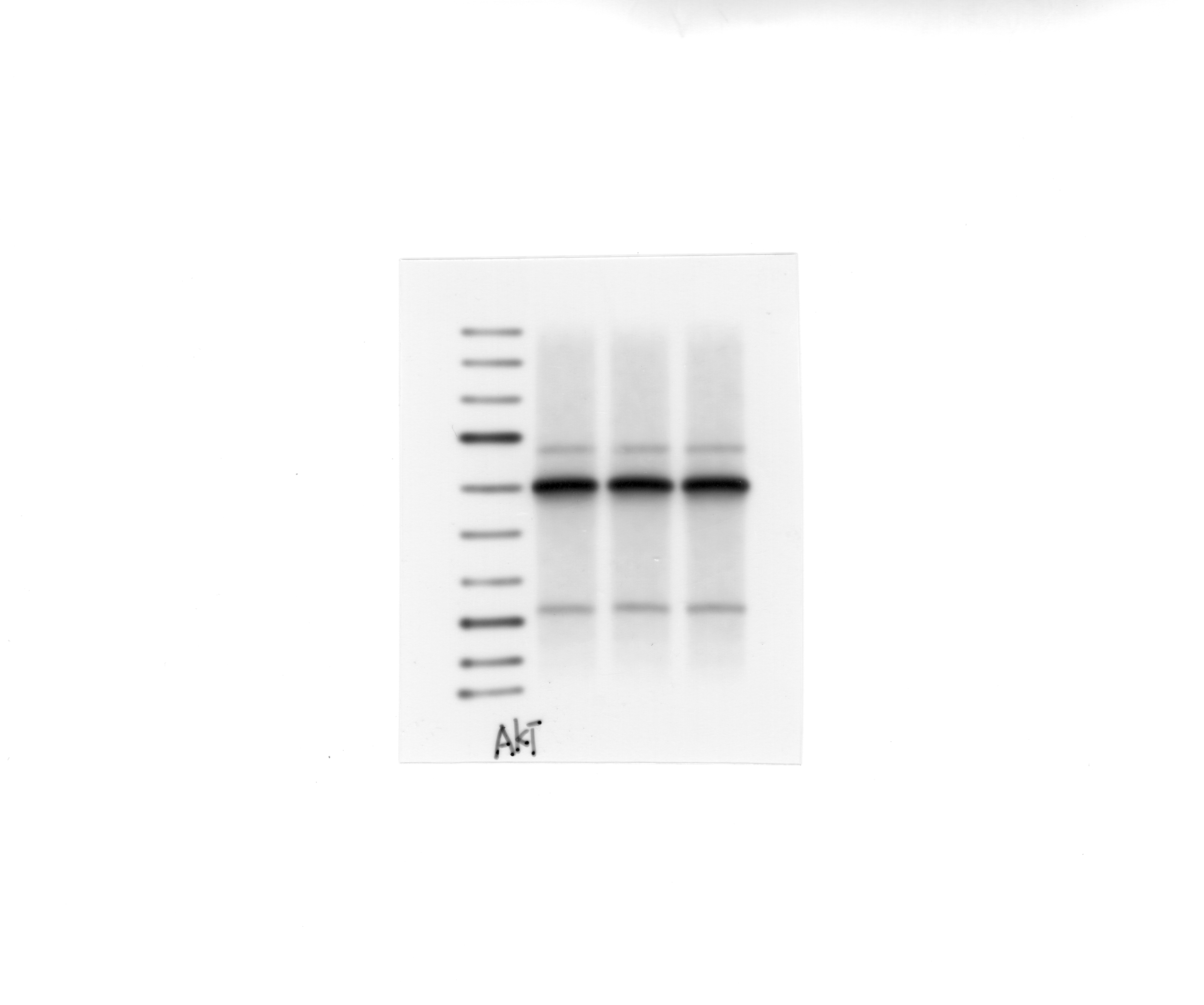

Supplement: Supplementary file 1 — Supplementary Information. [file 41598_2023_33792_MOESM1_ESM.zip › WB/fig 3B-SW480/AKT.tif]

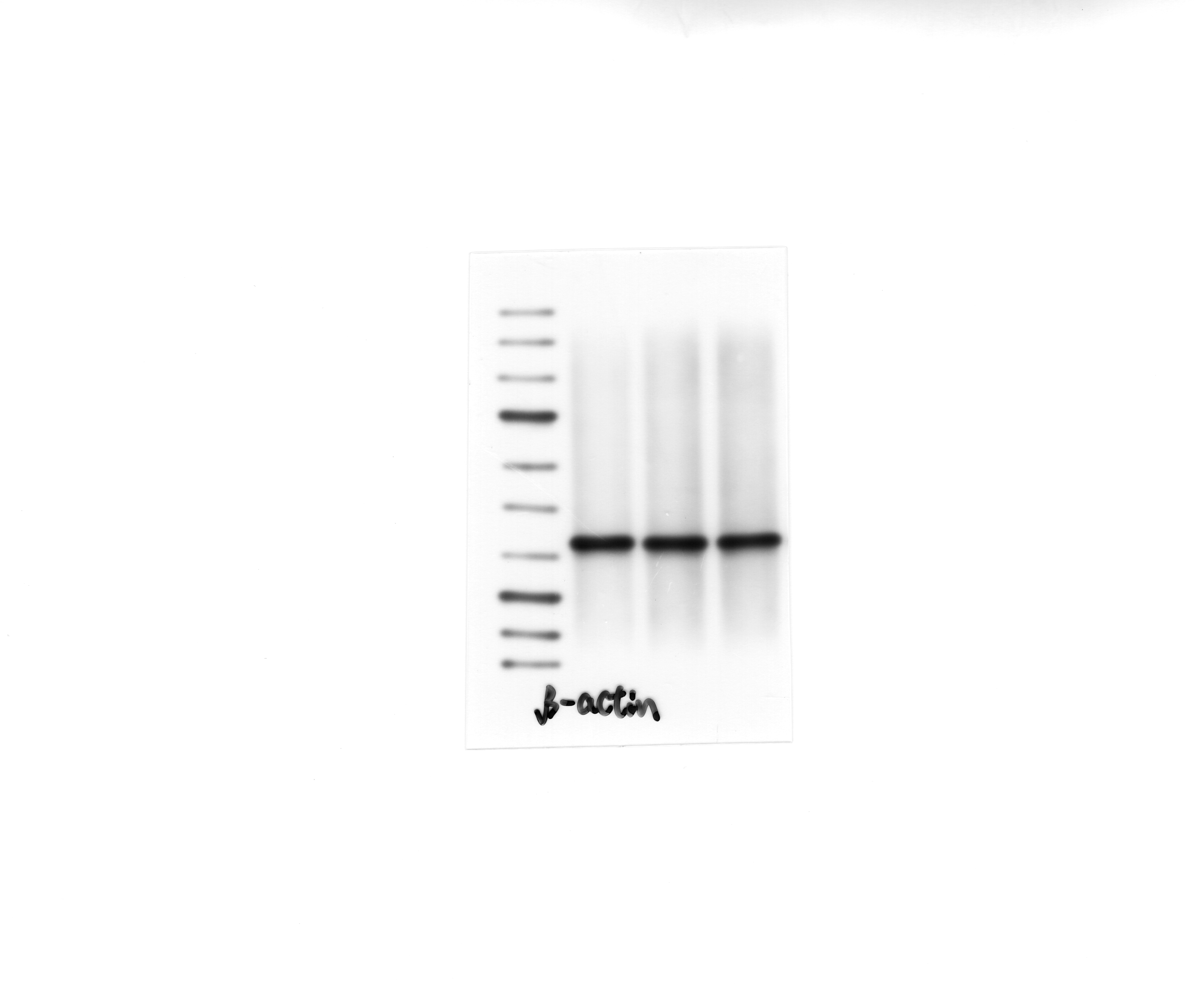

Supplement: Supplementary file 1 — Supplementary Information. [file 41598_2023_33792_MOESM1_ESM.zip › WB/fig 3B-SW480/Actin.tif]

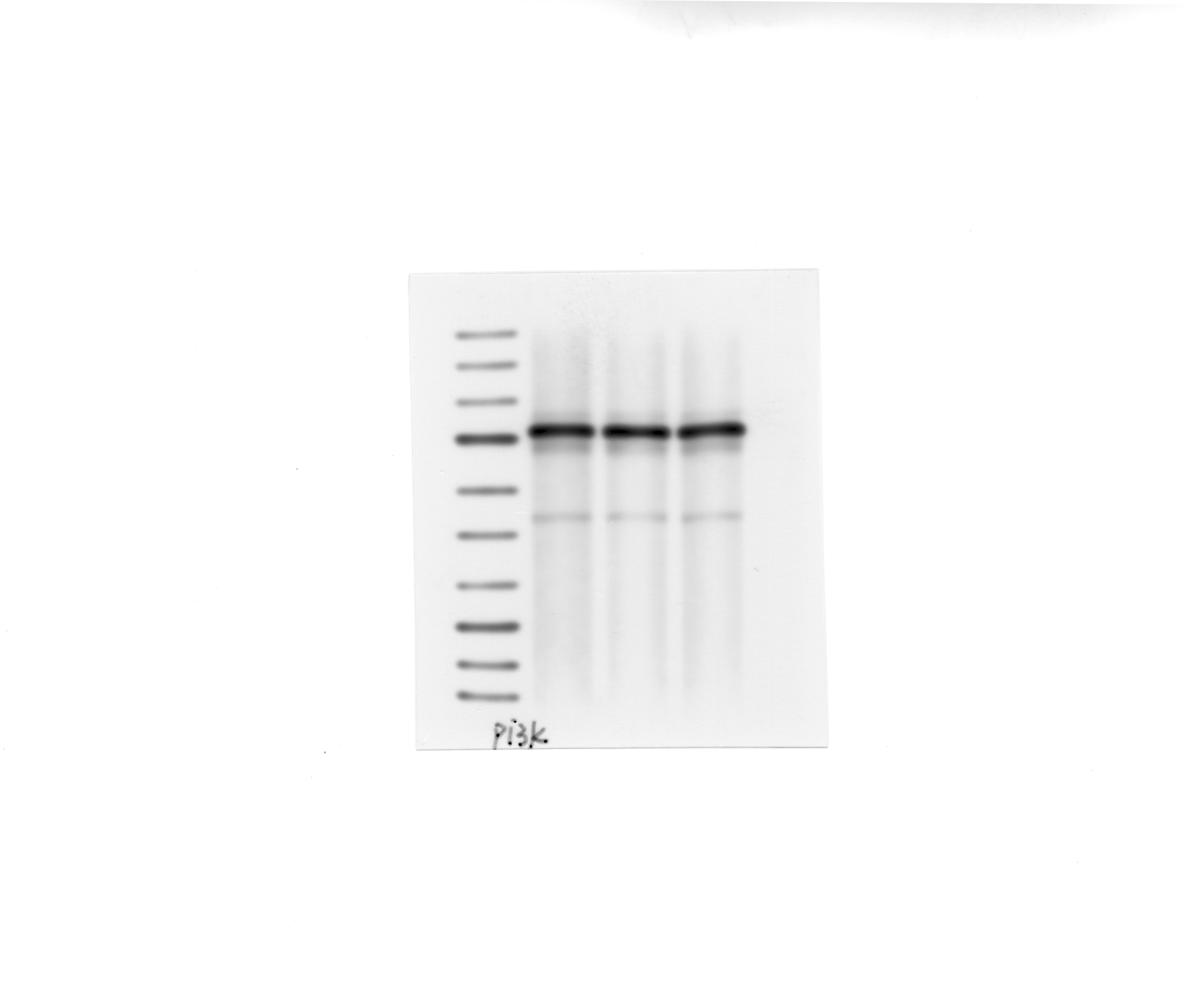

Supplement: Supplementary file 1 — Supplementary Information. [file 41598_2023_33792_MOESM1_ESM.zip › WB/fig 3B-SW480/PI3K.tif]

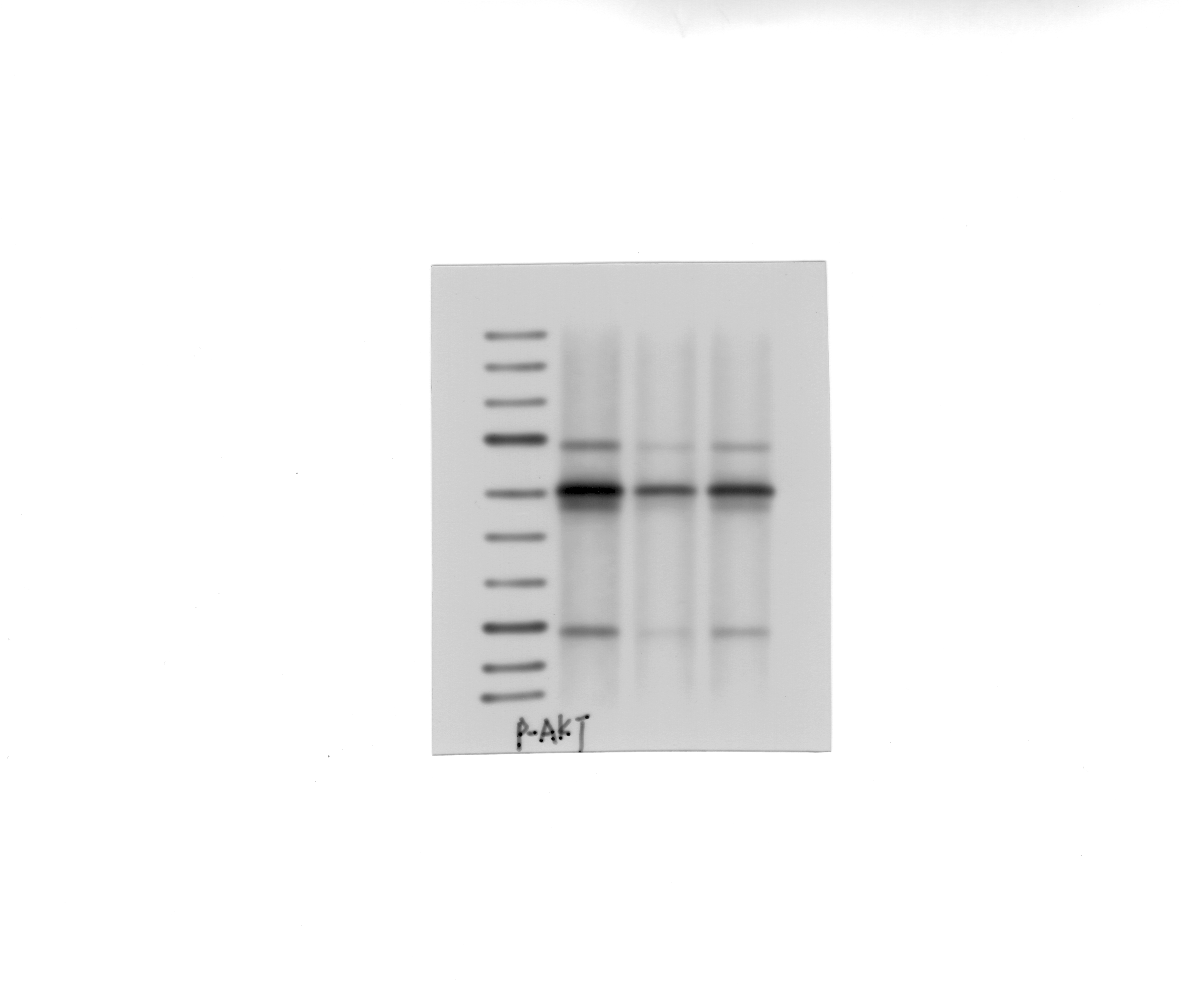

Supplement: Supplementary file 1 — Supplementary Information. [file 41598_2023_33792_MOESM1_ESM.zip › WB/fig 3B-SW480/p-AKT.tif]

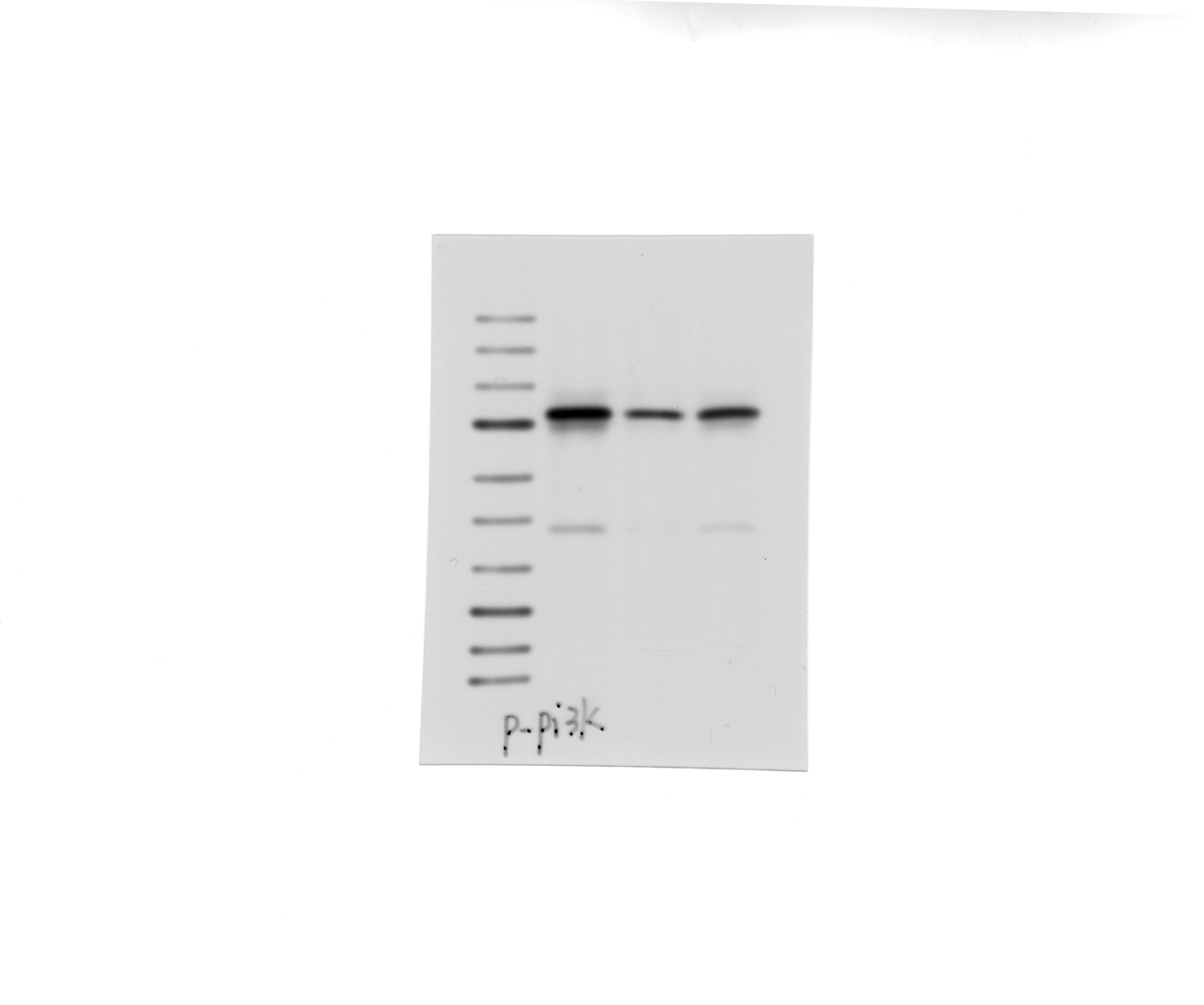

Supplement: Supplementary file 1 — Supplementary Information. [file 41598_2023_33792_MOESM1_ESM.zip › WB/fig 3B-SW480/p-PI3K.tif]

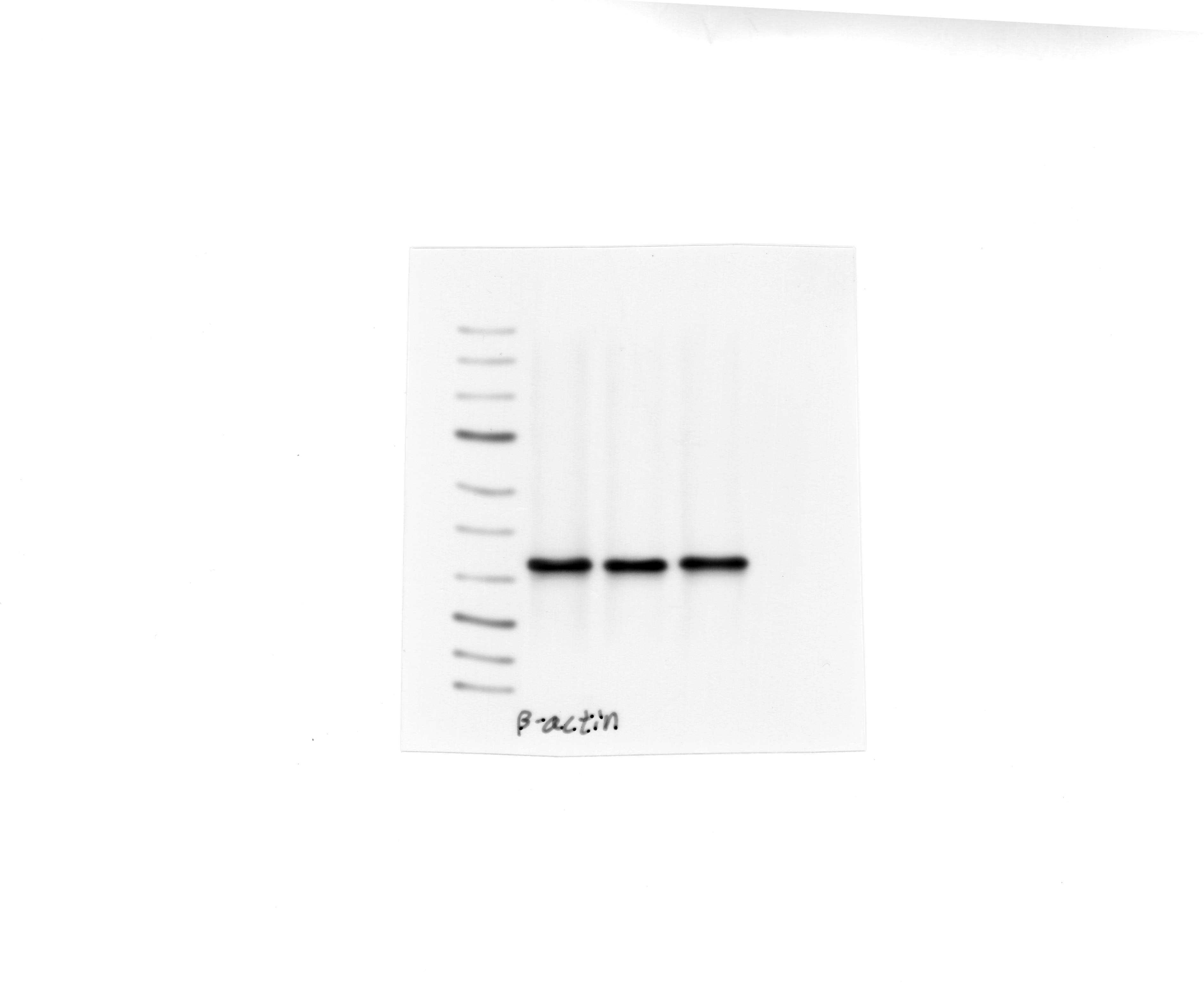

Supplement: Supplementary file 1 — Supplementary Information. [file 41598_2023_33792_MOESM1_ESM.zip › WB/fig 3H-HCT116/Actin.tif]

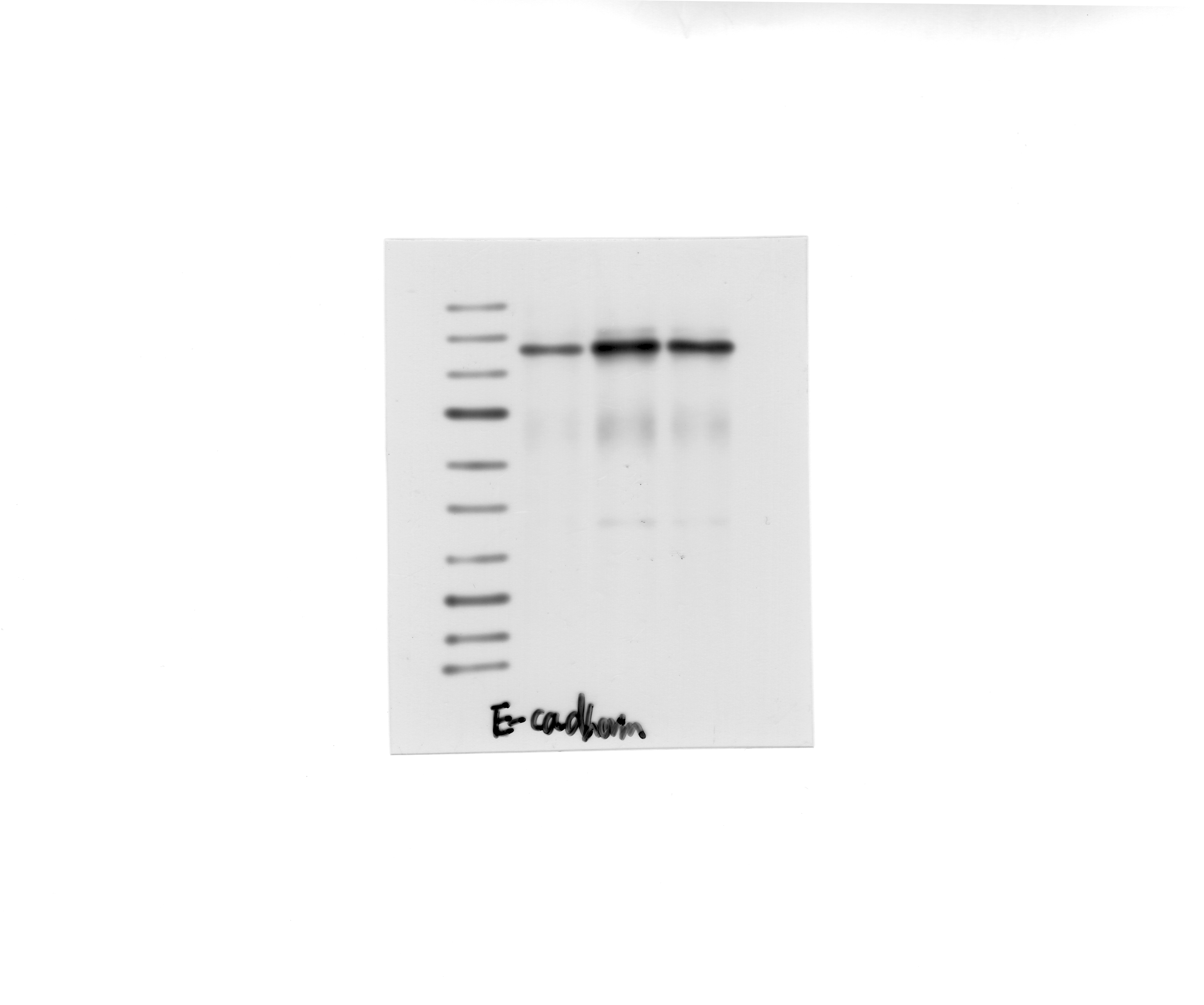

Supplement: Supplementary file 1 — Supplementary Information. [file 41598_2023_33792_MOESM1_ESM.zip › WB/fig 3H-HCT116/E-cadherin.tif]

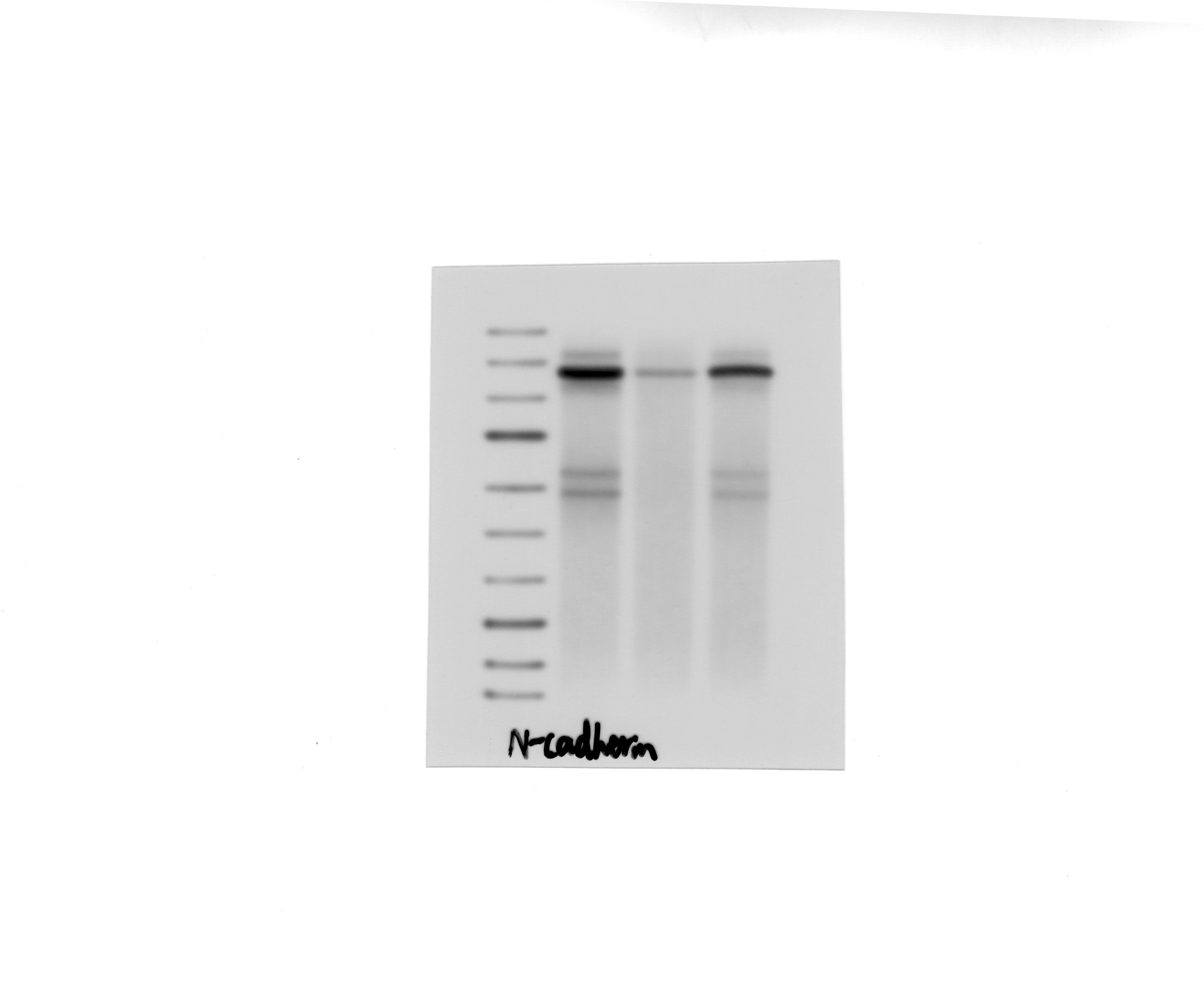

Supplement: Supplementary file 1 — Supplementary Information. [file 41598_2023_33792_MOESM1_ESM.zip › WB/fig 3H-HCT116/N-cadherin.tif]

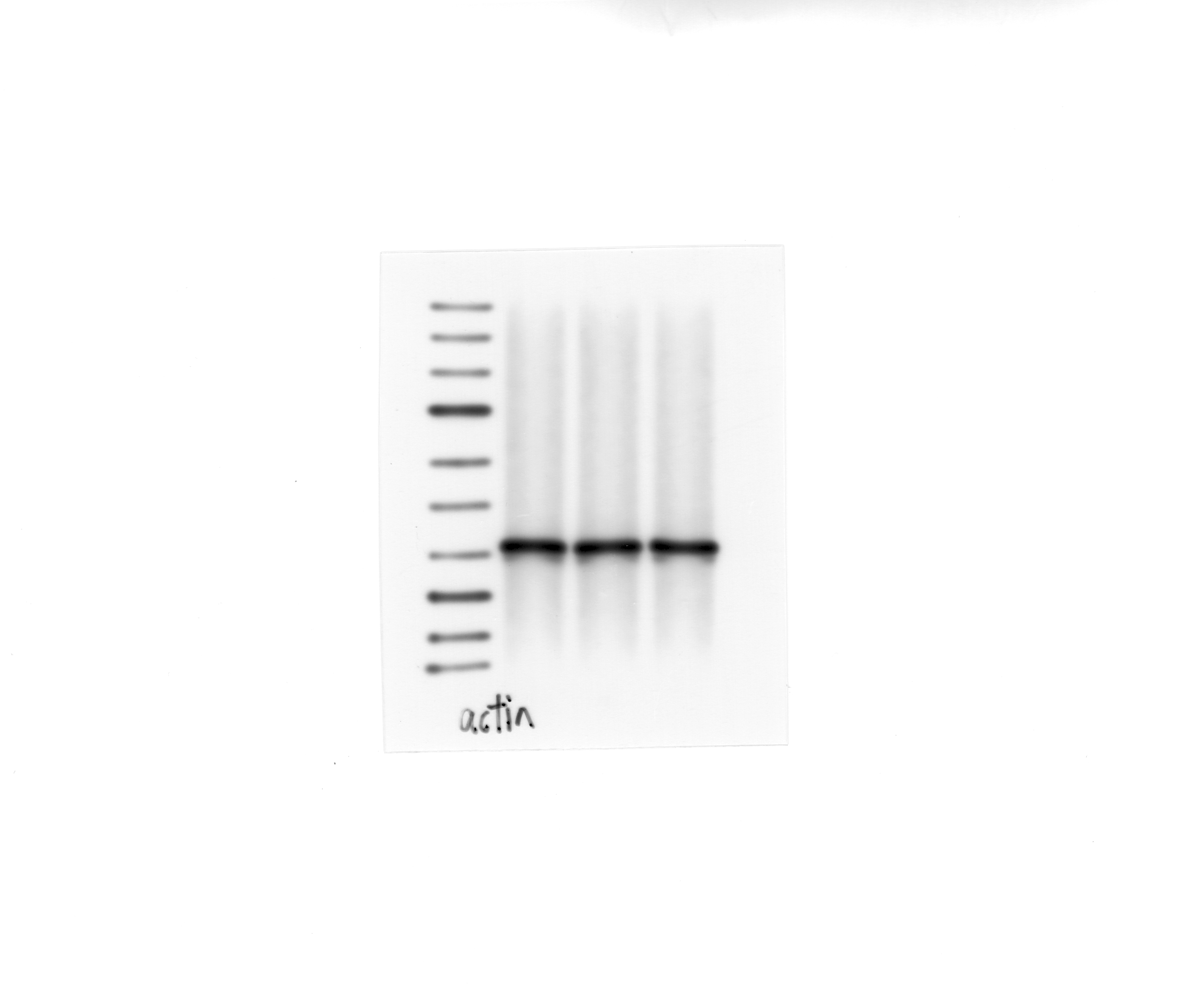

Supplement: Supplementary file 1 — Supplementary Information. [file 41598_2023_33792_MOESM1_ESM.zip › WB/fig 3H-SW480/Actin.tif]

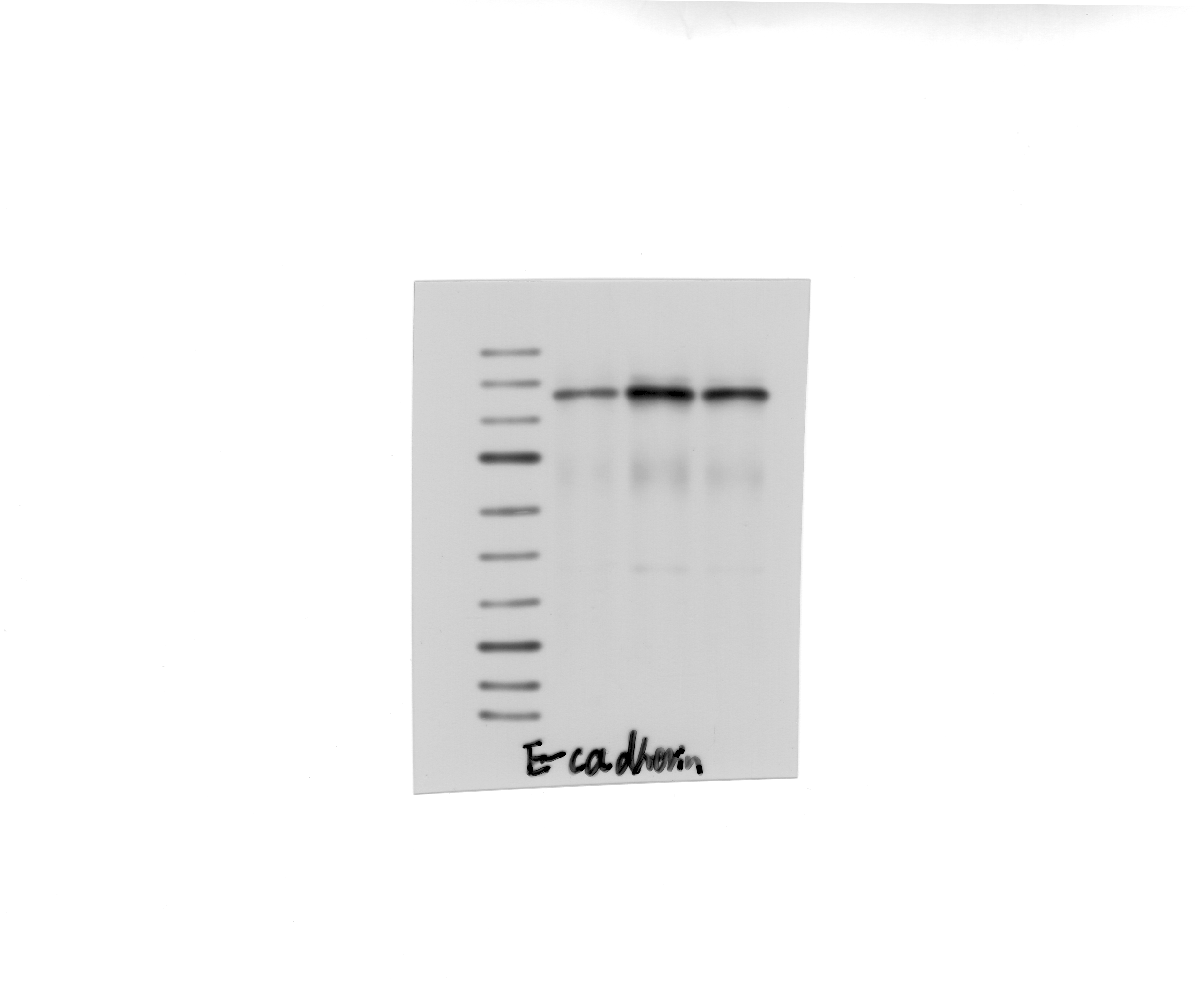

Supplement: Supplementary file 1 — Supplementary Information. [file 41598_2023_33792_MOESM1_ESM.zip › WB/fig 3H-SW480/E-cadherin.tif]

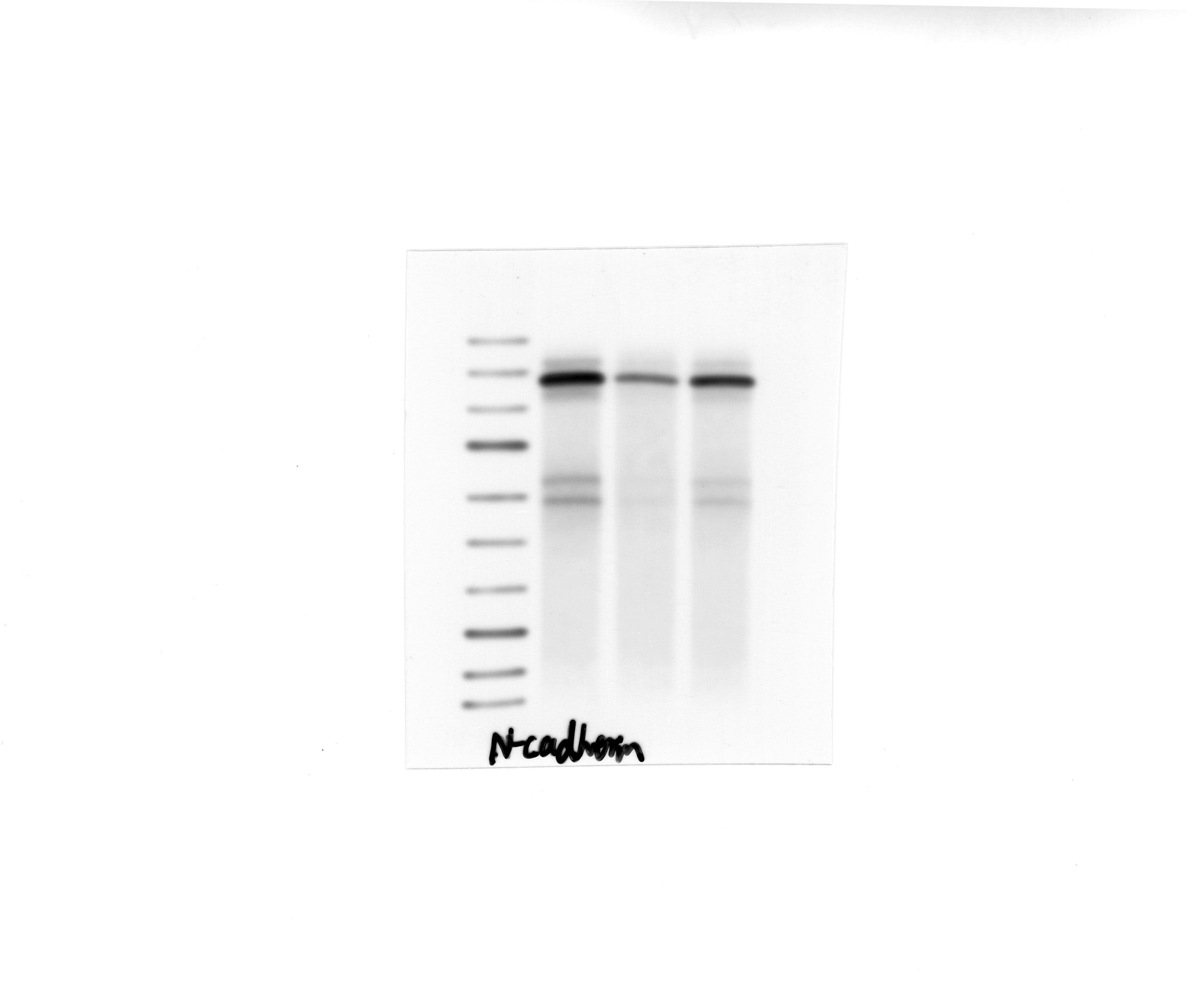

Supplement: Supplementary file 1 — Supplementary Information. [file 41598_2023_33792_MOESM1_ESM.zip › WB/fig 3H-SW480/N-cadherin.tif]
